# Selective CO_2_ electroreduction to methanol via enhanced oxygen bonding

**DOI:** 10.1038/s41467-022-35450-8

**Published:** 2022-12-15

**Authors:** Gong Zhang, Tuo Wang, Mengmeng Zhang, Lulu Li, Dongfang Cheng, Shiyu Zhen, Yongtao Wang, Jian Qin, Zhi-Jian Zhao, Jinlong Gong

**Affiliations:** 1 School of Chemical Engineering and Technology; Key Laboratory for Green Chemical Technology of Ministry of Education, Tianjin University, Tianjin, 300072 China; 2 Collaborative Innovation Center of Chemical Science and Engineering (Tianjin), Tianjin, 300072 China; 3 Haihe Laboratory of Sustainable Chemical Transformations, Tianjin, 300192 China; 4 Joint School of National University of Singapore and Tianjin University, International Campus of Tianjin University, Binhai New City, Fuzhou, 350207 China

**Keywords:** Nanoscale materials, Materials for energy and catalysis, Catalytic mechanisms

## Abstract

The reduction of carbon dioxide using electrochemical cells is an appealing technology to store renewable electricity in a chemical form. The preferential adsorption of oxygen over carbon atoms of intermediates could improve the methanol selectivity due to the retention of C–O bond. However, the adsorbent-surface interaction is mainly related to the *d* states of transition metals in catalysts, thus it is difficult to promote the formation of oxygen-bound intermediates without affecting the carbon affinity. This paper describes the construction of a molybdenum-based metal carbide catalyst that promotes the formation and adsorption of oxygen-bound intermediates, where the *sp* states in catalyst are enabled to participate in the bonding of intermediates. A high Faradaic efficiency of 80.4% for methanol is achieved at −1.1 V vs. the standard hydrogen electrode.

## Abstract (web summary)

While the reduction of CO_2_ to specific products offers a valuable approach mitigating gas emissions, it is challenging to control the formation of crucial intermediates. Here, authors report a strategy to promote the formation of oxygen-bound intermediates and boost the methanol production.

## Introduction

The high selective CO_2_ reduction reaction (CO_2_RR) driven by renewable electricity is a promising technology to realize carbon neutrality^1,2^. To improve the selectivity of CO_2_RR, it is a particularly effective method to adjust the adsorption energy and configurations of intermediates (i.e., C_*x*_H_*y*_O_*z*_) on the catalyst surface. Recently, various effective and enlightening strategies have been demonstrated, such as regulating catalysts with specific facets or low coordination sites^3,4^, alloying catalysts with foreign elements^5,6^, and modifying catalysts with organic molecules^7,8^. Sargent and co-workers. modified copper (Cu) surface with organic molecules to stabilize the atop-bound CO (CO_atop_) intermediate with the ratio of CO_atop_ to bridge-bound CO (CO_bridge_) controlled to make the coupling between CO_atop_ and CO_bridge_ as the lowest energy pathway, realizing a 72% Faradaic efficiency (FE) of ethylene under neutral media^9^. It is worth noting that the selective generation of oxygenates such as methanol (CH_3_OH) particularly relies on the adsorption energy and configurations of intermediates. This is because the corresponding rate-determining step (RDS) typically requires the participation of protons^10,11^. If the intermediate is bonded to the metal surface with its carbon atom, its oxygen atom will be positioned away from the surface, which makes protons from the electrolyte tend to attack this oxygen atom with a much higher possibility, resulting in the loss of oxygen atom and the decrease of oxygenates selectivity^12^. To promote the selective production of the oxygenate like CH_3_OH, the surface of the catalyst should exhibit appropriately oxophilic properties, thereby inducing the intermediates to be absorbed on the surface partially or totally through oxygen atoms to reduce the probability of oxygen loss. However, according to the *d*-band theory proposed by Nørskov et al., the binding of adsorbate on transition metal surface involves the interaction of both the *d* states and *sp* states of the transition metal, but the latter one barely shows any contribution to the change of bonding energy^13^. To some extent, such a scenario leads to the existence of a positive correlation between the carbon and oxygen adsorption energies^14^, which limits the catalyst to exhibit an oxophilic property while showing little effect on carbon affinity to avoid oxygen loss.

In this work, we describe a strategy to hybrid *d* states of metal with the *s* states of carbon, which promotes a significant change in *sp* states of the resulting catalysts during the adsorbate-surface bonding change process^14^, making it possible to avoid the interdependence between the carbon and oxygen adsorption energies^15–17^. Molybdenum (Mo)-based carbides nanoparticles were chosen as the candidate catalysts, as Mo sites were shown to stabilize C_*x*_H_*y*_O_*z*_ intermediates during CO_2_ hydrogenation^18,19^. Density functional theory (DFT) calculations and in-situ infrared spectroscopy are used to confirm the preferred formation of intermediates with oxygen-bound configurations. Finally, a new possible CH_3_OH formation pathway could be postulated alongside the traditional CO pathway, characterized by the presence of formyl (*OCHO*, an oxygen-bound species) as a representative intermediate, which could avoid further loss of oxygen atoms from the intermediates to promote the formation of CH_3_OH. This work provides an additional dimension to tune the scaling to design better catalysts.

## Results

### Characterizations of the Mo-based carbide catalyst

**Fig. 1:**
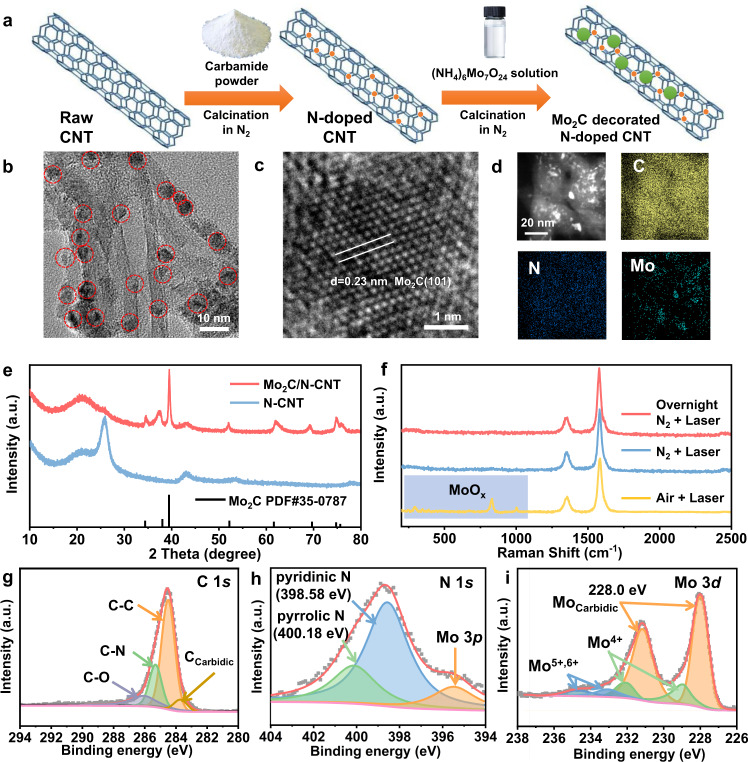
Morphologies and chemical states for Mo_2_C/N-CNT. **a** Schematic illustration for the preparation of Mo_2_C/N-CNT. **b** The TEM and **c** HRTEM images of Mo_2_C/N-CNT. **d** The dark-field scanning transmission electron microscopic (STEM) image and the corresponding elemental mapping results of C, N, and Mo of Mo_2_C/N-CNT. **e** XRD patterns and **f** Raman spectra of Mo_2_C/N-CNT and control samples. XPS of **g** C 1*s*, **h** N 1*s,* and **i** Mo 3*d* of Mo_2_C/N-CNT. Ar^+^ etching: 1000 eV for 100 s. The a.u. stands for arbitrary units.

To reduce the agglomeration of particles and increase the number of active sites, the particles were loaded onto the nitrogen-doped carbon nanotubes (N-CNT), using the strong metal-support interaction between Mo and N sites to drive the spatial dispersion of the particles^20^. In the synthesis process (Fig. 1a), the N sites were first introduced into the CNT by heating the mixture of raw CNT and carbamide under N_2_. Subsequently, Mo-based carbide nanoparticles were anchored on the N-CNT by heating the obtained N-CNT under the N_2_ atmosphere, in which ammonium molybdate serves as the metal source. Afterward, in situ surface passivation was performed at room temperature using 1 vol% O_2_. According to the relevant literature, low concentrations of O_2_ are able to dissociate on the Mo-based carbide surface at room temperature to form adsorbed oxygen which forms a passivation layer to avoid bulk oxidation during potential environmental exposure^21,22^. In addition, inert gas was used for sample protection during sample transfer and storge (details in “Methods”). Transmission electron microscopy (TEM) indicates the formation of uniformly dispersed particles with about 5.0 nm diameter anchored on N-CNT (Fig. 1b and Supplementary Fig. 1). A high-resolution TEM (HRTEM) image reveals that the lattice fringes with d-spacing of 0.23 and 0.26 nm, which correspond to the (101) and (100) plane of the hexagonal Mo_2_C (β-Mo_2_C), respectively (Fig. 1c), while no distinct lattice corresponding to the oxides appear (MoO_3_(100) d = 0.39 nm, MoO_2_(100) d = 0.48 nm and MoO_2_(011) d = 0.34 nm). Thus, the obtained catalyst is referred as to Mo_2_C/N-CNT. The elemental mapping (Fig. 1d) further suggests the well-defined spatial distribution of C, Mo, and N in the Mo_2_C/N-CNT. The structure of the Mo_2_C/N-CNT was also verified by powder X-ray diffraction (XRD). Aside from the peak from CNT, the obvious diffractions were all corresponded to the β-Mo_2_C (PDF#35–0787, P63/mmc, Fig. 1e). The absence of the nitride peak (PDF#04-003-2158) indicates that the Mo precursor did not react with the previously introduced N sites (Supplementary Fig. 2). Meanwhile, no diffraction peak of obvious oxides was observed in XRD (MoO_3_ PDF#05-0508, MoO_2_ PDF#32-0671), which is consistent with the TEM results. Further, Raman spectroscopy (Fig. 1f) using a 532 nm laser (illumination area ~ 7.9 × 10^−7^ mm^2^) of about 4 mW was repeated in different atmospheres (N_2_ or air), which excludes the presence of oxides in the catalyst (details in “Methods”). In the N_2_ atmosphere, the Raman bands of oxides were not observed. In the atmosphere of air, the bands of oxides were observed, along with the production of Mo^4+^ and Mo^6+^ visible in the Mo 3*d* X-ray photoelectron spectroscopy (XPS) result (Supplementary Fig. 3). Further, another freshly prepared sample upon passivation was placed in the air overnight and then tested under N_2_, which presented the absence of detectable oxides signal. This suggests that the catalyst might undergo noticeable bulk oxides formation when exposed to a high concentration of O_2_ (e.g., air) with the presence of an additional energy supply (e.g., laser or high temperature).

In order to perform a detailed analysis of the catalyst structure with high surface sensitivity, additional XPS characterizations were performed. The high-resolution C 1*s* XPS result (Fig. 1g) of Mo_2_C/N-CNT can be deconvoluted into five peaks locating at the binding energy of 283.2 eV (C_Carbidic_), 284.5 eV (C–C/C=C bonding), 285.2 eV (C–N), 286.3 eV (C–O–C bonding). The N 1*s* XPS result shows the presence of the pyridinic-N (380.0 eV), pyrrolic-N (399.3 eV) implying N-doping, and the pyridine N is the main species (Fig. 1h). Besides, the peaks located at 350.0 eV can be ascribed to the N associated with metal (N–Mo bond) and Mo 3*p*. Particularly, the binding energy of N 1 *s* in Mo_2_C/N-CNT negatively shifted compared with that in the bare N-CNT (Supplementary Fig. 4). This shift can be rationalized by the strong electron interactions of N and Mo_2_C^23^, which reveals the close contact between the N sites and the Mo_2_C particles. In order to resolve the oxidation state of the surface Mo, XPS upon Ar^+^ etching was performed. After the Ar^+^ etching, the fitting results of XPS signal from Mo_2_C/N-CNT reveals contributions from Mo^5+^ (or Mo^6+^) and Mo^4+^, while the primary component is carbidic Mo. The Mo^5+^ (or Mo^6+^) and Mo^4+^ species may originate from oxides that have not been etched (Supplementary Fig. 5)^24,25^. These oxides could result from unavoidable oxidation during sample transfer or from excessive oxidation during sample passivation. The XPS result of the freshly passivated sample (without Ar^+^ etching) exhibits more obvious oxides species (Supplementary Fig. 6). After semi-quantification of O and Mo (using the Avantage software), the atomic ratio of O to Mo before Ar^+^ etching (around 1:1) is found to be much lower than that in the Mo-based oxides (i.e., MoO_2_, MoO_3_), indicating that the passivation operation was able to keep the surface dominated by Mo_2_C during subsequent use. A comparison of Raman and XPS results shows that XPS is more suitable for detecting trace oxides species on the carbides surface. These characterization results indicate that a stable Mo-based compound has been formed that can be used in the subsequent CO_2_ reduction process.

### Promoted CH_3_OH production via Mo-based carbide

**Fig. 2:**
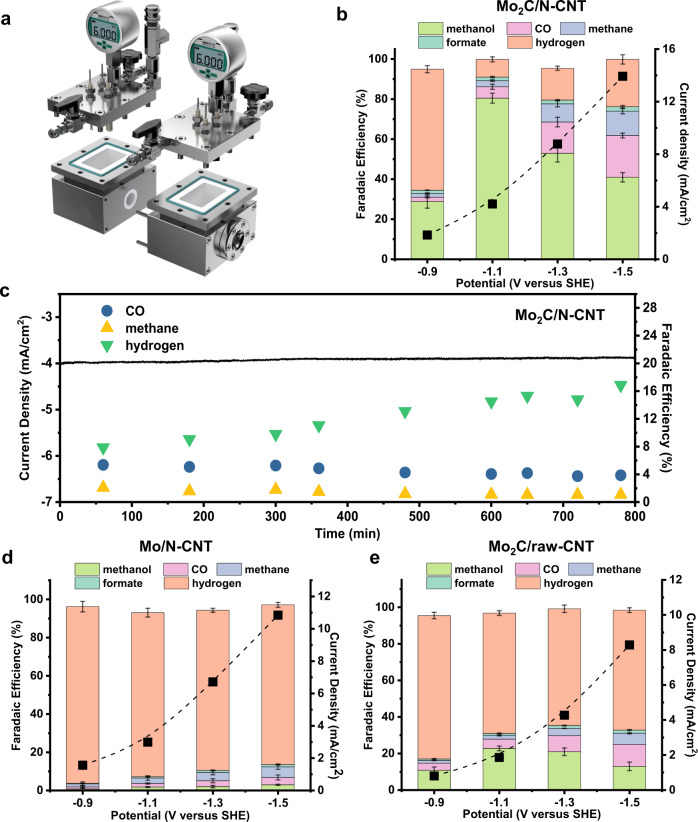
CO_2_RR performances of Mo_2_C/N-CNT and control samples. **a** Schematic of the high-pressure CO_2_RR system with 0.1 M KHCO_3_ as electrolyte. **b** Product distribution of Mo_2_C/N-CNT after 3 h CO_2_RR under 40 atm CO_2_ at various potentials. **c** Current–time (*I*–*t*) curves of Mo_2_C/N-CNT under the temporal evolution at the potential of −1.1 V vs. SHE for 800 min. Product distribution afforded by **d** Mo/N-CNT and **e** Mo_2_C/raw-CNT after 3 h CO_2_RR at various potentials under 40 atm CO_2_. Error bars represent the standard deviation from at least three independent measurements.

**Fig. 3:**
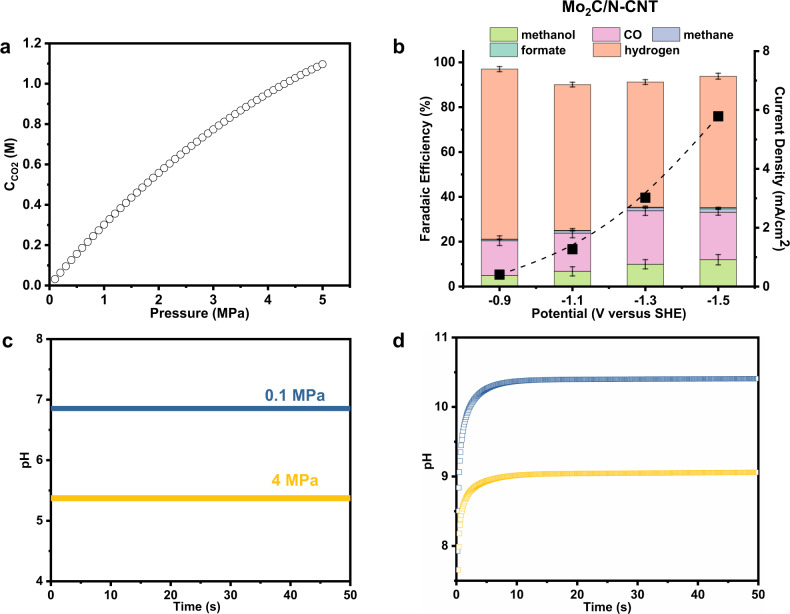
Investigation of the effect of CO_2_ pressure on catalyst performance. **a** The CO_2_ (aq) concentrations in 0.1 M KHCO_3_ under different pressures at 25 °C. **b** Product distributions of 3 h CO_2_RR with 0.1 M KHCO_3_ as electrolyte under 1 atm at various potentials. Two-dimensional plots of the time-dependent pH (under the potential of −1.1 V vs. SHE) at **c** the bulk electrolyte and **d** the electrode surface with CO_2_ pressures varying from 0.1 MPa (1 atm) to 4 MPa (40 atm). Error bars represent the standard deviation from at least three independent measurements.

The solubility of CO_2_ in the electrolyte under ambient pressure is only 0.033 M, thus it is difficult to supply sufficient CO_2_ to the catalyst, which makes it difficult to inhibit the hydrogen evolution reaction (HER). To supply sufficient CO_2_ in the slightly acidic electrolyte, a high-pressure continuous bubbling CO_2_ reduction system was used in this work (Fig. 2a and Supplementary Figs. 7, 8, details in Supplementary Text), which is expected to increase the surface coverage of CO_2_ and related intermediates. According to numerical simulation results (details in Supplementary Text), solubility of CO_2_ reached 0.95 M under 40 atm (4 MPa, Fig. 3a). CO_2_ molecules dissolved in water will form H_2_CO_3_ that can dissociate to obtain protons, thus an increasing amount of dissolved CO_2_ will lower the bulk and local pH of the electrolyte (Fig. 3c, d). For example, the bulk pH changed from 6.8 at 1 atm CO_2_ pressure to 5.4 at 40 atm CO_2_ pressure (Fig. 3c).

The CO_2_RR was then performed using a three-electrode configuration with 40 atm CO_2_ pressure (details in “Methods”, some typical gas chromatography, and ^1^H-NMR spectra are shown in Supplementary Figs. 9–11). CH_3_OH is the dominant liquid product under all potentials (Fig. 2b, typical calculations of ^1^H-NMR quantification are provided in Supplementary Text). As the potential decreases to −1.1 V vs. the standard hydrogen electrode (SHE, corresponding to −0.8 V vs. the reversible hydrogen electrode (RHE)), the selectivity of CH_3_OH reaches 80.4% (corresponding to a turnover frequency of 2.2 × 10^−2^ s^−1^ and a turnover number of 238.6, assuming all the supported Mo_2_C particles as active sites). At high overpotentials, the selectivity of CH_4_ gradually increased, indicating that it is easier to generate CH_3_OH than CH_4_ on the Mo_2_C/N-CNT surface under high CO_2_ pressure conditions (vide infra). Such a catalyst exhibits a high selectivity for CH_3_OH together with a reasonably large current density (>4 mA/cm^2^), which is an improved performance compared with previously reported systems for CH_3_OH production at ambient temperature and pressure (Supplementary Table 1). Compared with other well-designed Mo-based catalysts^26–28^, the catalyst synthesized in this work is a heterogeneous catalyst, which can provide abundant non-isolated sites for intermediates adsorption. At the same time, an aqueous electrolyte was used to maximize the availability of protons needed for the continuous hydrogenation of intermediates to CH_3_OH.

However, the selectivity of CH_3_OH decreases significantly at ambient pressure (Fig. 3a), which may be due to the decrease of local concentration (surface coverage) of active carbon species (i.e., CO_2_, CO) at ambient pressure (Supplementary Figs. 12, 13). According to the DFT calculations (vide infra), the Mo_2_C/N-CNT surface exhibits a low *CO desorption free energy (Supplementary Table 2). When the reaction pressure decreases, *CO desorbs easily, leading to an increase in the selectivity of CO. During the 800 min test under 40 atm pressure, the current density of Mo_2_C/N-CNT remained almost unchanged but was accompanied by a gradual increase in H_2_ selectivity (Fig. 2c). In addition, the average CH_3_OH production rate corresponding to different reaction times was gradually decreasing (Supplementary Fig. 14) with the final CH_3_OH FE (68.5%) being nevertheless higher than 80% of the optimal value (80.4%). The TEM results (Supplementary Figs. 15, 16) show that the crystal structure of the used Mo_2_C/N-CNT catalysts remains unchanged, but the content of the Mo element is reduced. Such results indicate that the decrease in stability mainly originates from the exfoliation of the particles. Enhancing the binding between the particles and CNT substrate is needed to improve the catalytic stability in the future.

Control experiments were further performed to ascertain that CH_3_OH was electrochemically formed on Mo_2_C sites through CO_2_ reduction. Firstly, N-CNT supported metallic Mo particles were prepared (referred to as Mo/N-CNT, details in “Methods”). The TEM image shows the existence of uniformly dispersed Mo particles with a d-spacing of 0.22 nm, corresponding to the Mo(110) plane (Supplementary Fig. 17). Catalytic performance tests using pure CO_2_ at 40 atm show that the high CH_3_OH selectivity could not be obtained without Mo_2_C as the main product of Mo/N-CNT is hydrogen (Fig. 2d). Besides, the contribution of the N sites to CH_3_OH formation could be eliminated based on the fact that bare N-CNT only produces CO as the carbonaceous product (Supplementary Fig. 18), which is consistent with previous reports^29^. At the same time, the extremely high HER performance of raw CNT prevents it from producing CH_3_OH (Supplementary Fig. 19). However, once the Mo_2_C particles are loaded on the raw CNT (details in “Methods”), the CH_3_OH selectivity of this combination (referred to as Mo_2_C/raw-CNT) will increase as expected (Fig. 2e), even though the selectivity is limited by the agglomeration of Mo_2_C particles on the raw CNT due to the lack of N sites (Supplementary Fig. 20). The reduction of ^13^CO_2_ was also carried out using the Mo_2_C/N-CNT catalyst. The results of ^1^H-NMR and ^13^C-NMR showed that only ^13^CH_3_OH was produced (Supplementary Figs. 21, 22), ruling out the contribution from impurities. In addition, no carbonaceous products were detected in control experiments where Ar was introduced as the only gas supply (Supplementary Fig. 23). These results suggest that CH_3_OH is mainly produced on the Mo_2_C sites in the Mo_2_C/N-CNT, while N sites promote dispersion and control the particle size of Mo_2_C. Notably, it is temporarily unable to provide clear evidence to determine whether the Mo–N interface acts as the active site for CO_2_ reduction to CH_3_OH in this study. Considering that the sample without N species still showed the ability to produce CH_3_OH, while the Mo_2_C sites are more abundant than Mo–N sites, it is speculated that the catalytic role of Mo_2_C might be greater than that of Mo–N. Mo–N models need to be constructed for theoretical calculations in the future work to predict the catalytic effect of the interface.

### Orbital hybridization assisted pathway control

**Fig. 4:**
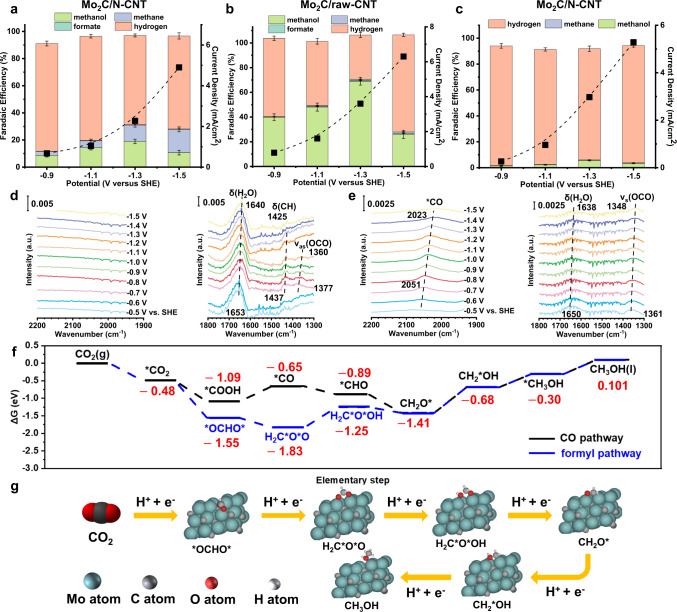
Mechanism exploration of the reaction of CO_2_ reduction to CH_3_OH. **a** Product distributions of Mo_2_C/N-CNT after 1 h CORR under 40 atm CO at various potentials (potassium phosphate buffer, pH ~ 5.4). **b** Product distributions of Mo_2_C/raw-CNT after 1 h CH_2_O reduction with 0.1 M potassium phosphate buffer (containing 100 mM CH_3_OH-free CH_2_O) as electrolyte under 40 atm Ar at various potentials. **c** Product distributions of Mo_2_C/N-CNT after 1 h HCOOH reduction under 40 atm Ar at various potentials. In-situ ATR-SEIRAS spectra of **d** Mo_2_C/N-CNT and **e** Mo/N-CNT. **f** The Gibbs free-energy diagrams of CO_2_ to CH_3_OH on the hydroxyl modified Mo_2_C/N-CNT surface at 0 V vs. RHE. **g** The favorable reaction intermediate configurations on the hydroxyl modified Mo_2_C/N-CNT surface, where the adsorbed hydroxyl is removed to clarify the adsorbate configurations. Error bars represent the standard deviation from at least three independent measurements. The a.u. stands for arbitrary units.

To explore the possible reaction pathways on the Mo_2_C/N-CNT surface, the CO reduction reaction (CORR) was first performed under 40 atm CO, which would bring a higher *CO coverage compared to that in CO_2_ reduction. However, the selectivity of CH_3_OH under CO reduction conditions is much lower than that in CO_2_RR (Fig. 4a). Meanwhile, the results of ^13^CO reduction verify that CH_3_OH is indeed derived from CO reduction (Supplementary Fig. 24). Such a phenomenon may result from the weak adsorption of *CO on the Mo_2_C/N-CNT surface and the low solubility of CO in electrolyte (ca. 1 mM at ambient pressure)^30^. For this reason, the desorption-free energy of *CO was calculated (Supplementary Table 2, the selection of the computational model is shown in the analysis below). It is found that the desorption-free energy of *CO on the surface of Mo_2_C/N-CNT is significantly reduced, which not favors the subsequent conversion of *CO on the Mo_2_C/N-CNT surface. Thus, it is possible that intermediates with stronger binding than *CO may indeed exist, which would potentially induce a new reaction pathway for CH_3_OH generation. According to the literatures, CH_2_O is another key intermediate for obtaining CH_3_OH. Next, a small amount (about 100 mM) of CH_2_O (CH_3_OH-free) was fed into the electrolyte to perform the CO_2_RR under high-pressure conditions^31^. Due to the presence of both Mo-based sites and N sites in the Mo_2_C/N-CNT sample, the interference of N sites on CH_2_O reduction was avoided by using raw CNT to support Mo_2_C^32^. Even though Mo_2_C particles may agglomerate on the raw CNT surface, this catalyst is still able to reduce CH_2_O to generate CH_3_OH as the main carbonaceous product (Fig. 4b). Considering that the selectivity of formate did not increase significantly, it indicates that the increased CH_3_OH selectivity indeed originates from the catalytic reduction reaction instead of the Cannizzaro disproportionation of CH_2_O^33^. The above results suggest that CH_2_O* may be an intermediate in the generation of CH_3_OH on the surface of Mo_2_C/N-CNT, which may present in both CO_2_ reduction and CO reduction processes. However, as the main competing product of CH_3_OH, the selectivity of CH_4_ showed a different trend as compared to that of CH_3_OH. Under the conditions of CO reduction, no significant loss of CH_4_ selectivity was observed, while the selectivity of CH_4_ was not promoted by the addition of CH_2_O. Therefore, in the process of CO_2_ reduction, the formation of CH_4_ on the surface of Mo_2_C/N-CNT may mainly undergo the CO pathway.

To explore the potential reaction intermediates on the Mo_2_C/N-CNT surface, in situ attenuated total reflectance-surface enhanced infrared absorption spectroscopy (ATR-SEIRAS) was further applied (details in “Methods”). In order to simulate the electrolyte pH under high pressure as much as possible, 0.1 M potassium phosphate buffer was used as the electrolyte (pH~5.9). The phosphate species are proven to not exhibit characteristic adsorption on the catalyst surface under reaction conditions and will not affect the pathway of CH_3_OH formation^34^. No obvious absorption peak of *CO stretching vibration is detected between 1800 and 2100 cm^−1^ on the Mo_2_C/N-CNT surface (Fig. 4d, left column), implying that the *CO binding on the Mo_2_C/N-CNT surface is not strong, which is consistent with the DFT calculation (Supplementary Table 2). However, three main peaks can be observed between 1300 and 1600 cm^−1^ (Fig. 4d, right column) on the Mo_2_C/N-CNT surface. The band at about 1437 cm^–1^ may be due to the asymmetric O–C–O stretching (*ν*_as_(OCO)) mode of bridge-bonded formate^35,36^, which is close to that predicted by our calculations (details in Supplementary Table 2), suggesting the presence of an *OCHO* intermediate on the Mo_2_C/N-CNT surface. The band at around 1377 cm^–1^ is assigned to the C–H bending (*δ*(CH)) vibration of the same species^35^. The broad peak locates around 1653–1640 cm^–1^ belongs to the bending mode of interfacial water. The infrared spectra of Mo/N-CNT are quite different from that of Mo_2_C/N-CNT. An obvious peak around 2051 cm^–1^ appears at −0.75 V vs. SHE (Fig. 4e, left column), which is assigned to the atop-bonded CO (CO_L_) on the Mo/N-CNT surface^37^ and the redshift of this band with the potential negatively shifting from −0.5 to −1.5 V vs. SHE is ascribed to the Stark tuning effect^38^. A single peak near 1361 cm^–1^ (Fig. 4e, right column) may correspond to the vibration of a species that rarely exists on the surface of Mo_2_C/N-CNT. According to the previous researchers, it is most likely derived from the symmetric O–C–O stretching (ν_s_(OCO)) mode of absorbed *COOH intermediate^35,39^, (details in Supplementary Table 3), which can selectively produce CO after the further proton-coupled electron transfer process. Further measurements at atmospheric pressure also show similar results, i.e., a decrease in *CO coverage and an increase in *OCHO* coverage on the Mo_2_C/N-CNT surface (Supplementary Figs. 26, 27). Thus, the IR spectral results illustrate that formyl might exhibit a higher coverage on the Mo_2_C/N-CNT surface than on the Mo surface, suggesting that CO_2_ could obtain CH_3_OH via the formyl intermediate (referred as formyl pathway), which can be attributed to the possible oxophilic nature of the catalyst introduced by the formation of Mo-based carbides.

Theoretical calculations were further performed to accurately obtain the adsorption energies and adsorption configurations of the intermediates. According to previous studies, oxides do not possess CO_2_ reduction activities, thus the oxides present on the surface are spectators^40^. In addition, considering that oxides are rarely present on the surface, they are unlikely to be the main active center. Since the Mo_2_C/N-CNT surface features surface adsorbed oxygen after passivation, hydroxyl (*OH) might be present on the Mo_2_C/N-CNT surface under the reaction conditions of aqueous solution^41^, despite the absence of oxides. According to our calculations, we found that under the reaction conditions (−0.1 V vs. SHE), the most stable hydroxyl coverage of the surface is 1/8 ML (Supplementary Fig. 28, detailed analysis can be found below). It is noteworthy that the calculated hydroxyl coverage is lower than that measured by XPS (the atomic ratio of O to Mo is around 1:1), probably due to the unavoidable oxidation during sample transfer and oxygen loss under reduction conditions, thus in-situ (operando) characterizations are highly required in the future. Therefore, bare Mo_2_C(101), hydroxyl modified Mo_2_C(101), and bare Mo(110) were used as model structures for calculation (the extended Mo_2_C slab model can be found in Supplementary Fig. 29). In terms of thermodynamics (Fig. 4f, g and Supplementary Figs. 30, 31), the hydroxyl modified Mo_2_C surface (related to Mo_2_C/N-CNT) prefers to absorb *OCHO* intermediate (bidentate adsorption configuration with two oxygen atoms bound on the surface) rather than the *COOH intermediate (atop adsorption configuration with only one carbon atom bound on the surface), thus CO_2_ prefers to convert to *OCHO* at beginning of the reaction. In addition, according to the results of in situ ATR-SEIRAS and DFT calculations, *CO may easily desorb from the Mo_2_C/N-CNT surface (i.e., the hydroxyl-modified Mo_2_C(101)), which is not favorable for subsequent conversions. After the *OCHO* formation, it can be reduced to obtain the H_2_C*O*OH intermediate, which undergoes further reaction to yield CH_2_O* and CH_3_OH^33^. In this regard, the reduction of HCOOH was carried out in the Ar atmosphere at 40 atm to explore the presence of H_2_C*O*OH (potassium phosphate buffer with 100 mM HCOOH, pH~5.4)^42^. Although CH_3_OH was obtained (Supplementary Fig. 32), the selectivity of CH_3_OH was rather low (Fig. 4c), probably due to the low concentration of free HCOOH molecules in the electrolyte at such pH conditions.^13^C-labeled HCOOH reduction verified that the detected trace amounts of CH_3_OH were indeed from *HCOOH reduction (Supplementary Fig. 33). Further calculations also confirm that HCOOH molecules can be adsorbed on the surface and converted to H_2_C*O*OH (Supplementary Table 4). However, the high thermodynamic potential for CO_2_ reduction to HCOOH hinders the production of HCOOH during CO_2_ reduction (Supplementary Fig. 34). Thus, H_2_C*O*OH should be an intermediate for CO_2_ reduction and is mainly obtained from the stepwise hydrogenation of *OCHO*. Considering that CH_4_ is the most important competing product of CH_3_OH, the free energies to obtain CH_4_ and CH_3_OH were also compared (Fig. 4f and Supplementary Figs. 30, 34). According to previous reports, CH_4_ obtained via the CO pathway may involve intermediates such as *CHO, *CH, and *CH_2_^10^. Our calculations show that the predicted limiting potentials (*U*_L_, the onset potential) of CH_4_ is higher (*U*_L_ = −1.04 V vs. RHE) than the hydrogenation of CH_2_O* to obtain CH_2_*OH (*U*_L_ = −0.73 V vs. RHE), explaining the experimental phenomenon that CH_4_ is less selective than CH_3_OH. The higher free energy to CH_3_OH production on the bare Mo_2_C surface (Supplementary Figure 35) compared to the hydroxyl modified Mo_2_C, along with its lower *U*_L_ for CH_4_ production (*U*_L_ = −0.90 V vs. RHE) than for CH_3_OH production (*U*_L_ = −1.21 V vs. RHE), confirms our hypothesis that the active phase is the hydroxyl-terminated surface. This could be due to the transfer of electrons from Mo sites to hydroxyl groups, resulting in a lower electron density at the Mo site. Specifically, Bader charge calculations suggesting a 0.65 *e* transfer of Mo site to hydroxyl. Mo sites with decreased electron density weaken the adsorption to key intermediates, thus tunes the thermodynamic potential of the elementary reaction step (Supplementary Table 5). However, on the bare Mo surface, due to the high free energy for H_2_C*O*O formation (Supplementary Fig. 36), it is difficult for Mo to generate CH_3_OH from CO_2_RR through the formyl pathway. However, it is worth noting that although the current calculations have provided much valuable information, more precise electrolyte–electrode interface models still need to be developed to gain deeper insights.

Further, the enhancement of oxygen bonding on the Mo_2_C/N-CNT surface was also emphasized by using single atom oxygen adsorbents (*O) as the probe for calculation. As would be expected, the oxygen bonding strength (Supplementary Fig. 27) on the Mo_2_C surface is significantly improved upon the hybridization between Mo and carbon atoms, which is favorable to enhance the adsorption energy of intermediates bound by oxygen atoms, therefore promoting the formation of such intermediates, avoiding the loss of oxygen atoms and elevating the selectivity of oxygenates (e.g., CH_3_OH). Moreover, to verify the possibility of *OH presence under the reaction conditions, we have calculated the free energy of *O to *OH and *OH to H_2_O, respectively, which indicates that *OH could be readily generated under electrochemical conditions (Supplementary Fig. 27).

In order to verify the existence of hybridization and to rationalize the oxophilic property due to hybridization, the electronic structure (density of states, DOS) of Mo_2_C was also calculated. Unlike the similarity of the metal projected *d* states between Mo_2_C and Mo, the metal projected *sp* states of the Mo_2_C undergo a significant transition from the parent Mo (Supplementary Fig. 37), i.e., the appearance of a dominant splitting of the Mo_2_C *sp* states into bonding states around −10 eV. Thus, the splitting of Mo_2_C *sp* states suggests the existence of hybridization between Mo *d* and carbon *s* states^14^, making possible a significant contribution of Mo_2_C *sp* states in the change of adsorbate-surface binding energy, which explains the enhanced oxygen bonding of Mo_2_C, consistent with the previous findings^14^.

## Discussion

In summary, this work proposes a strategy to control the adsorption energy of CO_2_ reduction intermediates using metal-carbon hybridization, which facilitates the formation and conversion of oxygen-bound intermediates by enhancing the adsorption energy of oxygen atoms. According to the calculation of electronic structure, the hybridization between the metal (Mo) *d* states and the carbon *s* states is supported by the *sp* states splitting of the resulting catalysts (Mo_2_C). This hybridization allows the *sp* states to work with *d* states in the control of the adsorption of intermediates. In situ ATR-SEIRAS combined with theoretical calculations suggest the potential existence of a new CH_3_OH formation pathway, i.e., CH_3_OH may be obtained via the formyl pathway (determined by oxygen-bound intermediates) alongside the CO pathway (determined by oxygen-bound intermediates), thus facilitating the conversion of CO_2_ to CH_3_OH. Finally, following the optimization of the adsorption configurations of the intermediates, the FE of CH_3_OH reached 80.4%. From a thermodynamic point of view, this work presents a new approach to activate the *sp* states of catalysts via the hybridization of different electron states, which could realize tunable control of the adsorption mode of intermediates to inhibit extra oxygen loss for enhanced oxygenates generation. In addition, through increasing the reaction pressure, the coverage of CO_2_ and *CO on the catalyst surface was elevated to facilitate the reaction efficiency. However, it is worth clarifying that the strong oxygen binding leads to high thermodynamic barrier in some reaction steps (CH_2_O* → CH_2_*OH), thus a more negative applied potential is required. Future optimization of intermediate adsorption through element doping may be needed. Since the formyl pathway is an effective pathway that may inhibit CH_4_ production, the conversion of CO_2_ to formyl would be hindered if *CO occupies excessive sites. Therefore, the reaction pressure also deserves further optimization. It is particularly noteworthy that the formyl pathway still requires more experimental and computational evidence than the currently well-accepted CO pathway. For example, future kinetic isotope effects could be explored, time-resolved vibrational spectroscopy should be used, more accurate electrode–electrolyte interfaces need to be established, and reaction kinetic barriers should be calculated. In conclusion, this paper provides an initial result that exhibits a key step to overcome the bottleneck in the electrical production of oxygenated products.

## Methods

### Sample preparation

Activation of the raw carbon nanotube (CNT): 2 g CNT was firstly dispersed in 200 mL of 60 wt.% HNO_3_ solution under ultrasonication for 60 min. Then, the refluxing was carried out in an oil bath at 120 °C for 9 h. Finally, the turbid liquid was centrifuged, washed with ultra-purity water to neutral pH, and dried at 60 °C.

Synthesis of the N doped CNT (N-CNT): The treated CNT was mixed with urea with a mass ratio of 1:10, and then heated up in a tube furnace to 800 °C under a gas flow of 80 standard cubic centimeters per minute (sccm) N_2_ and maintained for 1 h, obtaining the final N-CNT.

Synthesis of the Mo_2_C anchored N-CNT (Mo_2_C/N-CNT): In a typical synthetic process, N-CNT suspension was prepared by adding 0.1 g N-CNT to 100 mL deionized water with the aid of sonication for 1 h, and then 10 mL ammonium molybdate aqueous solution (2.5 mg/mL) was added dropwise into N-CNT suspension under the vigorous stirring overnight and then centrifuged to collect the products and dried at 60 °C. The as-prepared powder was further calcined at 600 °C for 2 h under a gas flow of 80 sccm N_2_. The obtained black power was Mo_2_C/N-CNT. After cooling down, the sample was in-situ passivated at room temperature in a flow of 1 vol % O_2_ in N_2_ for 0.5 h (250 mL/(min·g_cat_))^22,43^. After synthesis, the sample was transferred within 30 s to an Ar-filled sample tube, which was then transferred to an Ar-filled self-sealing bag and sequentially wrapped into four additional Ar-filled self-sealing bags (each step was completed within 30 s). The above wrapped samples were then quickly transferred to the glove box. The samples were stored in a glove box. The Mo_2_C/raw-CNT was obtained using the same synthesis method except replacing N-CNT with CNT.

Synthesis of Mo anchored N-CNT (Mo/N-CNT):^20^ In a typical synthetic process, N-CNT suspension was prepared by adding 0.1 g N-CNT to 100 mL deionized water with the aid of sonication for 1 h, and then 10 mL ammonium molybdate aqueous solution (2.5 mg/mL) was added dropwise into N-CNT suspension under the vigorous stirring overnight and then centrifuged to collect the products and dried at 60 °C. The as-prepared powder was further calcined at 800 °C for 8 h under a gas flow of 100 sccm H_2_. The obtained black power was Mo/N-CNT. The samples were then quickly transferred to a glove box for storage according to the transfer method described above.

Preparation of the catalysts ink: Typically, 10 mg of sample and 40 μL of Nafion solution (5 wt%, Dupont) are dispersed in 5 mL of ethanol solution in a glove box. Then it is rapidly transferred to the Ultrabath Sonicator (LTB-500), and the well-dispersed ink is obtained by sonication for 1 h.

Preparation of the working electrode: 500 µL of the ink was then drop casted onto a 1.5 × 1 cm^2^ polytetrafluoroethylene-treated carbon paper (Toray TGP-H-060, ~190 μm, Fuel Cell Store) to cover an area of 1 × 1 cm^2^ (catalyst mass loading, 0.5 mg cm^−2^). The prepared electrodes were fully dried using an infrared lamp. The above steps are all performed in the glove box. Finally, the prepared electrodes were quickly assembled into the reactor.

Preparation of the 0.1 M KHCO_3_ electrolyte: For the CO_2_ reduction, a 0.05 M K_2_CO_3_ solution was firstly prepared using 18.2 MΩ ultra-purity water and K_2_CO_3_ powder. Metallic impurities in the as-prepared solution were then removed by chelating the solution with Chelex® 100. Finally, this solution was bubbled with pure CO_2_ at a rate of 10 sccm for at least 30 min to obtain the final 0.1 M KHCO_3_ electrolyte.

For the Ar reduction, a 0.1 M KHCO_3_ solution was prepared using 18.2 MΩ ultra-purity water and KHCO_3_ powder. Metallic impurities in the as-prepared solution were then removed by chelating the solution with Chelex® 100.

For the ^13^CO_2_ reduction, 0.05 M ^13^C-labeled K_2_CO_3_ was used as the electrolyte. This solution was bubbled with pure ^13^CO_2_ at a rate of 10 sccm for at least 30 min to obtain the final 0.1 M ^13^C-labeled KHCO_3_ electrolyte.

### Characterizations

TEM, High-resolution TEM (HRTEM) images were obtained at 200 kV (JEOL JEM-2100F). The crystal structure was determined by X-ray Diffractometer (XRD, Bruker D8 Focus) with Cu Kα radiation (λ = 1.54056 Å) at 40 kV and 40 mA. XRD spectra were collected over a 2θ range of 10–80° at a scanning speed of 8°/min XPS analyses of precatalysts were carried out on a Thermo Scientific™ K-Alpha+ system with an Al Kα X-ray source (1486.6 eV). For the XPS test, a vacuum transfer module (Thermo Scientific™) was used for moving air-sensitive samples from a glove box to the system. The binding energy was calibrated using the C 1*s* photoelectron peak at 284.8 eV as the reference. Raman spectroscopy was performed with a confocal Raman microscopy system (LabRAM HR Evolution, Horiba Jobin Yvon) with a Linkam’s CCR1000 stage. A 532 nm laser served as the excitation source. An air objective (Olympus MPlan N, 50×, numerical aperture = 0.75) was used for measurements. The laser power was about 4 mW. Each presented spectrum is an average of 6 continuously acquired spectra with a collection time of 10 s each.

In-situ ATR-SEIRAS measurements: In-situ ATR-SEIRAS was performed with an ATR configuration. Au nanofilms were deposited directly on the reflecting plane of a Si prism using a modified electroless chemical deposition method outlined by Xu et al.^44^. The catalyst was sprayed onto the surface of Au nanofilms. The spectroelectrochemical cell was home designed to suit an elevated reaction pressure (around 3 atm). Although the reaction pressure has deviated from the most suitable value for CH_3_OH formation, resulting in a decreased CH_3_OH product selectivity (the Mo_2_C/N-CNT still shows enhanced CH_3_OH production performance than the Mo/N-CNT), the adsorption of critical intermediates on the catalysts surface is still present, thus the ATR-SEIRAS could still be used to detect intermediates on different surfaces. The counter electrode (a graphite rod) was separated from the working and reference electrodes, i.e., the catalyst film and a home-designed saturated Ag/AgCl electrode with a salt bridge (saturated KCl solution, Supplementary Fig. 38, customer designed), respectively, with a piece of a bipolar membrane (Fumasep FBM, Fumatech). The stirring effect within the cell is from the rotation of a magnetic stirring bar. This cell is integrated into the FTIR (is10, Nicolet) spectrometer with a modified accessory at a 60° incident angle (VeeMax III, PIKE Technology). All spectra were collected with a 4 cm^−1^ resolution. Spectra are presented in absorbance, with positive and negative peaks showing an increase and decrease in signal, respectively. The background was taken at open circuit potential in Ar saturated electrolyte for each electrode. Electrochemical measurements are carried out with a potentiostat (VERTEX. ONE. EIS, IVIUM).

### Analysis of Ar/CO_2_/CORR products

For the CO_2_RR, gas products were quantified using an online gas chromatography system (GC7890B, Agilent Technologies, Inc.). The thermal conductivity detector (TCD) connected to a MolSieve 5A packed column (Agilent Technologies, Inc.) to detect H_2_ and a flame ionization detector (FID, referred as to FID 1) connected to a Porapak Q packed column (Agilent Technologies, Inc.) to detect CO. A methanizer was installed to enable FID 1 to detect CO with 1000 times higher sensitivity. Another FID (referred as to FID 2) connected to an HP-PLOT Al_2_O_3_ capillary column (Agilent Technologies, Inc.) to detect hydrocarbons (C_2_H_4_ and C_2_H_6_). Ar was used as the carrier gas.

For the CORR, the same GC (GC7890B, Agilent Technologies, Inc.) is used, but the valve switching time and the temperature of the methanizer need to be optimized. In order to avoid the poisoning of the MolSieve 5A column and to avoid the coke formation in the methanizer, the methanizer temperature should be lowered to room temperature and the valve should be switched in advance for backflushing before the CO enters the MolSieve 5A column, thus enabling the detection of H_2_ using TCD and the detection of hydrocarbon products using the FID 2 (such an operation can make the FID 1 with almost no signal). The measurements were made over the course of seven GC injections (intervals of 720 s between adjacent injections). To ensure that the reported data is from a system under equilibrium conditions, only measurements obtained from the 4th to 7th injections were used for analyses.

The reduction of HCOOH/CH_2_O was carried out under Ar atmosphere. The highly volatile CH_2_O was cooled to 6 °C in a refrigerator, together with a pipettor. Then CH_2_O was added to the electrolyte with the cold pipettor of the same low temperature (~ 6 °C). The transfer of CH_2_O sample from the ampoule to the reactor was within 30 s to avoid the oxidization and polymerization of CH_2_O.

The quantitative analysis was conducted using calibration curves obtained by a series of standard gas mixtures (Messer Group). The standard gas uses CO_2_ as a balanced component. The mixture includes H_2_ (299.1 ppm, 1047 ppm, 18376.3 ppm, 2.01%), CO (103 ppm, 199.5 ppm, 994.9 ppm, 998.6 ppm), CH_4_ (20 ppm, 198.1 ppm, 500.4 ppm, 9917.4 ppm), C_2_H_4_ (20 ppm, 99.4 ppm, 487.5 ppm, 1001.6 ppm) and C_2_H_6_ (19.9 ppm, 99.0 ppm, 521.2 ppm, 968.2 ppm). The peak areas of the gas phase products were converted to mole concentration using calibration curves.

The liquid products were collected from the cathode and anode chambers after electrolysis and analyzed by headspace gas chromatography (HS-GC) and ^1^H-NMR. HS-GC measurements were carried out using a BCHP HS-2 Headspace Sampler with GC2060 gas chromatography (Shanghai Ruimin Instrument Co., Ltd.). Typically, 10 mL vials were filled with 3 mL of the liquid sample and sealed. They were heated to 70 °C over 20 min in the headspace sampler and 1 mL of the headspace gas composition was automatically injected into the GC. The sample loop (110 °C) and transfer line (110 °C) were both heated to avoid condensation. N_2_ was used as the carrier gas. An HP-INNOWax capillary column (Length: 60 m; ID: 0.32 mm; Film: 0.5 μm, Agilent) was used to separate the compounds in the sample. The peak area of methanol was converted to mole concentration using calibration curves that were obtained using methanol standard solution (10 mg/mL, purchased from Beijing Yihuatongbiao Tech. Co., Ltd., and has been certified by the China National Institute of Metrology) diluted with H_2_O to different concentrations (0.1, 1, 5, 10, 20 mM). ^1^H-NMR was performed using AVANCE III^TM^ HD 400 MHz NanoBAY with solvent (H_2_O) suppression. Inverse gated decoupling technology is used unless otherwise stated. 400 μL of electrolyte was mixed with 100 μL of a solution of 10 mM dimethyl sulfoxide (DMSO) and 50 mM phenol in D_2_O as internal standards for the ^1^H-NMR analysis. The internal standards, phenol and DMSO, were chosen because they did not interfere with peaks arising from CO_2_ reduction products and because of their non-volatility which allowed for use and storage of the same internal standards solution for all of the product measurements without appreciable change in concentration. The area of product peaks to the right of the water peak was compared to the area of DMSO (at a chemical shift of 2.6 ppm), and the area of product peaks to the left of the water peak was compared to the area of phenol (at a chemical shift of 7.2 ppm)^45^. To avoid the loss of highly volatile species (e.g., CH_3_OH), all electrolytes containing liquid products are stored in a refrigerator (2–8 °C).

An uncompensated resistance (*R*_u_) was determined by potentiostatic electrochemical impedance spectroscopy (EIS) and was compensated to 85% using the potentiostat. The EIS was operated under open-circuit voltage with a frequency ranging from 10^5^ to 0.01 Hz. All the reported cell voltages were corrected by the measured internal resistance loss under each specific test unless otherwise stated.

### Computational methods

Geometry optimization and DFT calculations were carried out with the Vienna Ab initio Simulation Package (VASP)^46^. The calculations employed the generalized-gradient approximation (GGA) in the form of the Bayesian error estimation functional with van der Waals corrections^47^. The cut-off energy is 400 eV. The interactions between the atomic cores and electrons were described by the projector augmented wave method^48^. Optimized geometries were found when the force on each relaxed atom was less than 0.02 eV·Å^–1^.

In our work, we used the β-hexagonal Mo_2_C phase, consistent with our XRD results (Fig. 1e). Considering that no uniform model for β-hexagonal Mo_2_C is available, we employed the structure which Jiao et al. proposed^49^. (3 × 3 × 1) Monkhorst-Pack grid K-points were used for (1 × 2) of Mo_2_C (101) with 4 layers and (4 × 4)-Mo (110) with 4 layers. Optimized geometries were found when the force on each relaxed atom was less than 0.02 eV·Å^–1^ (details in Supplementary Table 6). The free energy for intermediate was calculated as Δ*G* = Δ*E* + ΔZPE – *T*Δ*S*, where Δ*E* is the reaction energy change from DFT calculations, ΔZPE is the change of zero-point energies (ZPE) which is calculated with the vibrational frequencies of absorbates and molecules (details in Supplementary Table 7), and Δ*S* is the entropy change in the reaction^12^. *T* is temperature and is set to 298 K. For adsorbates, the entropy was calculated with Harmonic oscillator approximation (details in Supplementary Table 7)^12^. The computational hydrogen electrode (CHE) model was employed to determine free energies of intermediates. Solvation corrections are conducted according to the work of Calle-Vallejo and co-workers (details in Supplementary Table 8)^50^.

## Supplementary information

Supplementary Information

## Data Availability

The data generated in this study are provided in Supplementary Information and Source Data file. Source data are provided with this paper.

## Source data

Source Data

## Code availability

All codes are available upon reasonable request.

## References

[1] De Luna P (2019). What would it take for renewably powered electrosynthesis to displace petrochemical processes?. Science.

[2] Birdja YY (2019). Advances and challenges in understanding the electrocatalytic conversion of carbon dioxide to fuels. Nat. Energy.

[3] Arán-Ais RM, Scholten F, Kunze S, Rizo R, Roldan CB (2020). The role of in situ generated morphological motifs and Cu(i) species in C_2+_ product selectivity during CO_2_ pulsed electroreduction. Nat. Energy.

[4] Jiang K (2018). Metal ion cycling of Cu foil for selective C–C coupling in electrochemical CO_2_ reduction. Nat. Catal..

[5] Clark EL, Hahn C, Jaramillo TF, Bell AT (2017). Electrochemical CO_2_ reduction over compressively strained CuAg surface alloys with enhanced multi-carbon oxygenate selectivity. J. Am. Chem. Soc..

[6] Zhong M (2020). Accelerated discovery of CO_2_ electrocatalysts using active machine learning. Nature.

[7] Kim D (2020). Selective CO_2_ electrocatalysis at the pseudocapacitive nanoparticle/ordered-ligand interlayer. Nat. Energy.

[8] Nam DH (2020). Intermediate binding control using metal-organic frameworks enhances electrochemical CO_2_ reduction. J. Am. Chem. Soc..

[9] Li F (2020). Molecular tuning of CO_2_-to-ethylene conversion. Nature.

[10] Nie X, Esopi MR, Janik MJ, Asthagiri A (2013). Selectivity of CO_2_ reduction on copper electrodes: the role of the kinetics of elementary steps. Angew. Chem. Int. Ed..

[11] Kas R, Kortlever R, Yılmaz H, Koper MT, Mul G (2015). Manipulating the hydrocarbon selectivity of copper nanoparticles in CO_2_ electroreduction by process conditions. ChemElectroChem.

[12] Peterson AA, Abild-Pedersen F, Studt F, Rossmeisl J, Norskov JK (2010). How copper catalyzes the electroreduction of carbon dioxide into Hhydrocarbon fuels. Energy Environ. Sci..

[13] Li Y, Sun Q (2016). Recent advances in breaking scaling relations for effective electrochemical conversion of CO_2_. Adv. Energy Mater..

[14] Michalsky R, Zhang Y-J, Medford AJ, Peterson AA (2014). Departures from the adsorption energy scaling relations for metal carbide catalysts. J. Phy. Chem. C.

[15] Li Y, Reuter K (2020). Active-site computational screening: role of structural and compositional diversity for the electrochemical CO_2_ reduction at Mo carbide catalysts. ACS Catal..

[16] Hong X, Chan K, Tsai C, Norskov JK (2016). How doped MoS_2_ breaks transition-metal scaling relations for CO_2_ electrochemical reduction. ACS Catal..

[17] Chan K, Tsai C, Hansen HA, Nørskov JK (2014). Molybdenum sulfides and selenides as possible electrocatalysts for CO_2_ reduction. ChemCatChem.

[18] Liu P, Choi Y, Yang Y, White MG (2010). Methanol synthesis from H_2_ and CO_2_ on a Mo_6_S_8_ cluster: a density functional study. J. Phys. Chem. A.

[19] Liu C, Liu P (2015). Mechanistic study of methanol synthesis from CO_2_ and H_2_ on a modified model Mo_6_S_8_ cluster. ACS Catal..

[20] Chen J (2019). Redispersion of Mo-based catalysts and the rational design of super small-sized metallic Mo species. ACS Catal..

[21] Ovari L, Kiss J, Farkas AP, Solymosi F (2005). Surface and subsurface oxidation of Mo_2_C/Mo(100): low-energy ion-scattering, Auger electron, angle-resolved X-ray photoelectron, and mass spectroscopy studies. J. Phys. Chem. B.

[22] Marquart W (2021). CO_2_ reduction over Mo_2_C-based catalysts. ACS Catal..

[23] Jiao Y, Zheng Y, Chen P, Jaroniec M, Qiao S-Z (2017). Molecular scaffolding strategy with synergistic active centers to facilitate electrocatalytic CO_2_ reduction to hydrocarbon/alcohol. J. Am. Chem. Soc..

[24] Lim KRG (2020). 2H-MoS_2_ on Mo_2_CTx MXene nanohybrid for efficient and durable electrocatalytic hydrogen evolution. ACS Nano.

[25] Murugappan K (2018). Operando NAP-XPS unveils differences in MoO_3_ and Mo_2_C during hydrodeoxygenation. Nat. Catal..

[26] Mouchfiq A, Todorova TK, Dey S, Fontecave M, Mougel V (2020). A bioinspired molybdenum–copper molecular catalyst for CO_2_ electroreduction. Chem. Sci..

[27] Asadi M (2014). Robust carbon dioxide reduction on molybdenum disulphide edges. Nat. Commun..

[28] Handoko AD (2020). Two-dimensional titanium and molybdenum carbide MXenes as electrocatalysts for CO_2_ reduction. iScience.

[29] Wu J, Sharifi T, Gao Y, Zhang T, Ajayan PM (2019). Emerging carbon-based heterogeneous catalysts for electrochemical reduction of carbon dioxide into value-added chemicals. Adv. Mater..

[30] Li J (2019). Effectively increased efficiency for electroreduction of carbon monoxide using supported polycrystalline copper powder electrocatalysts. ACS Catal..

[31] Garza AJ, Bell AT, Head-Gordon M (2018). Mechanism of CO_2_ reduction at copper surfaces: pathways to C_2_ products. ACS Catal..

[32] Song Y (2017). Metal-free nitrogen-doped mesoporous carbon for electroreduction of CO_2_ to ethanol. Angew. Chem. Int. Ed..

[33] Low QH, Loo NWX, Calle-Vallejo F, Yeo BS (2019). Enhanced electroreduction of carbon dioxide to methanol using zinc dendrites pulse-deposited on silver foam. Angew. Chem. Int. Ed..

[34] Yang K, Kas R, Smith WA (2019). In situ infrared spectroscopy reveals persistent alkalinity near electrode surfaces during CO_2_ electroreduction. J. Am. Chem. Soc..

[35] Kattel S, Yan B, Yang Y, Chen JG, Liu P (2016). Optimizing binding energies of key intermediates for CO_2_ hydrogenation to methanol over oxide-supported copper. J. Am. Chem. Soc..

[36] Gao D (2017). Switchable CO_2_ electroreduction via engineering active phases of Pd nanoparticles. Nano Res..

[37] Zhao Y (2020). Speciation of Cu surfaces during the electrochemical CO reduction reaction. J. Am. Chem. Soc..

[38] Zhu S, Jiang B, Cai W, Shao M (2017). Direct observation on reaction intermediates and the role of bicarbonate anions in CO_2_ electrochemical reduction reaction on Cu surfaces. J. Am. Chem. Soc..

[39] Firet NJ, Smith WA (2016). Probing the reaction mechanism of CO_2_ electroreduction over Ag films via operando infrared spectroscopy. ACS Catal..

[40] Griesser C (2021). True nature of the transition-metal carbide/liquid interface determines its reactivity. ACS Catal..

[41] Tian X, Wang T, Jiao H (2017). Oxidation of the hexagonal Mo_2_C(101) surface by H_2_O dissociative adsorption. Catal. Sci. Technol..

[42] Zhu W (2020). Enhanced CO_2_ electroreduction on neighboring Zn/Co monomers by electronic effect. Angew. Chem. Int. Ed..

[43] Lin L (2017). Low-temperature hydrogen production from water and methanol using Pt/alpha-MoC catalysts. Nature.

[44] Dunwell M (2017). The central role of bicarbonate in the electrochemical reduction of carbon dioxide on gold. J. Am. Chem. Soc..

[45] Kuhl KP, Cave ER, Abram DN, Jaramillo TF (2012). New insights into the electrochemical reduction of carbon dioxide on metallic copper surfaces. Energy Environ. Sci..

[46] Kresse G, Hafne J (1994). Ab initio molecular-dynamics simulation of the liquid-metal-amorphous-semiconductor transition in germanium. Phys. Rev. B Condens. Matter.

[47] Wellendorff J (2012). Density functionals for surface science: exchange-correlation model development with Bayesian error estimation. Phys. Rev. B.

[48] Kresse G, Joubert D (1999). From ultrasoft pseudopotentials to the projector augmented-wave method. Phys. Rev. B.

[49] Wang T (2011). Stability of β-Mo_2_C facets from ab initio atomistic thermodynamics. J. Phy. Chem. C.

[50] Granda-Marulanda LP (2020). A semiempirical method to detect and correct DFT-based gas phase errors and its application in electrocatalysis. ACS Catal..

