# Supplementary material for: Selective CO2 electroreduction to methanol via enhanced oxygen bonding
Source: Nat Commun. 2022 Dec 15;13:7768. doi: 10.1038/s41467-022-35450-8 (PMC9755525; doi:10.1038/s41467-022-35450-8)
Supplement: Supplementary file 1 — Supplementary Information [file 41467_2022_35450_MOESM1_ESM.pdf]

Supplementary Information

Selective CO_2 Electroreduction to Methanol via Enhanced Oxygen Bonding

Gong Zhang,[‡,a,b,c] Tuo Wang, [‡,a,b,c,d] Mengmeng Zhang,[‡,a,b,c] Lulu Li,[a,b,c] Dongfang Cheng,[a,b,c] Shiyu Zhen,[a,b,c] Yongtao Wang,[a,b,c] Jian Qin,[a,b,c] Zhi-Jian Zhao,[a,b,c] and Jinlong Gong[*,a,b,c]

[a]School of Chemical Engineering and Technology; Key Laboratory for Green Chemical Technology of Ministry of Education, Tianjin University, Tianjin 300072, China.

[b]Collaborative Innovation Center of Chemical Science and Engineering (Tianjin), Tianjin 300072, China.

[c]Haihe Laboratory of Sustainable Chemical Transformations, Tianjin 300192, China

[d]Joint School of National University of Singapore and Tianjin University, International Campus of Tianjin University, Binhai New City, Fuzhou 350207, China.

‡These authors contributed equally to this work.

*e-mail: jlgong@tju.edu.cn

Contents

1. Experimental Section

2. Supplementary Figures and Tables

3. Supplementary Text

4. References

Experimental Section

Materials

The multiwall carbon nanotube was purchased from Chengdu Organic Chemicals Co. Ltd. Ammonium molybdate (99.999%-Mo) was purchased from Real & Lead Chemical Co., LTD. HNO_3 (AR) was purchased from Damao Chemical Reagent Factory, Tianjin. Urea (AR), ethanol (GC), K_2CO_3 (AR), phenol (GC), DMSO (GC), D_2O (99.96 atom% D), $KHPO_4$ (99.99%), KH_2PO_4 (99.99%), KOH (99.999%), Chelex® 100 (Na form), $H^{13}COOH$ (99 atom % ^{13}C), $K_2{}^{13}CO_3$ (98 atom % ^{13}C) were purchased from Sigma-Aladdin. Methanol-free formaldehyde (16% w/v, Pierce™) was purchased from Thermo Fisher. The reagents were used without any purification process.$^{13}CO_2$ and ^{13}CO were purchased from Global Rare Gases, LLC. Ultra-purity water (18.25 MΩ·cm) was supplied by the Merck Milli-Q Direct Q5 system. CO_2, CO, Ar, H_2, N_2 and 1% O_2 + N_2 mixed gas all supplied by Air Liquide (≥ 99.999%).

Supplementary Figures and Tables

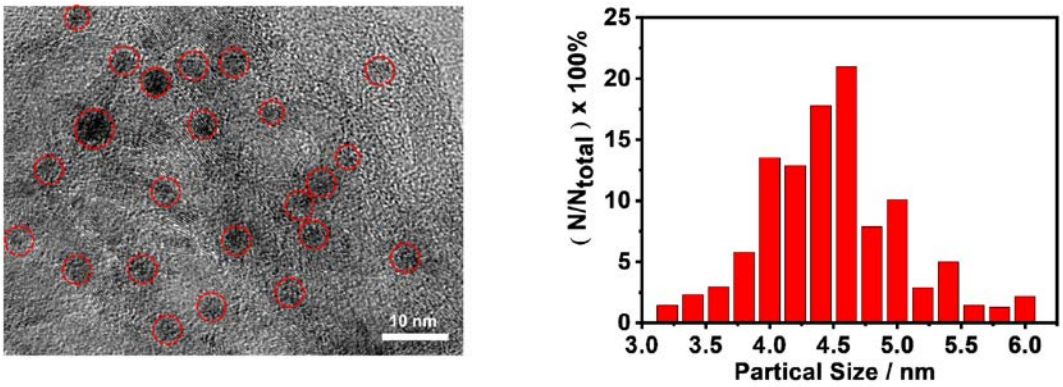

Supplementary Fig. 1 Size distribution of Mo_2C nanoparticles on N-CNT.

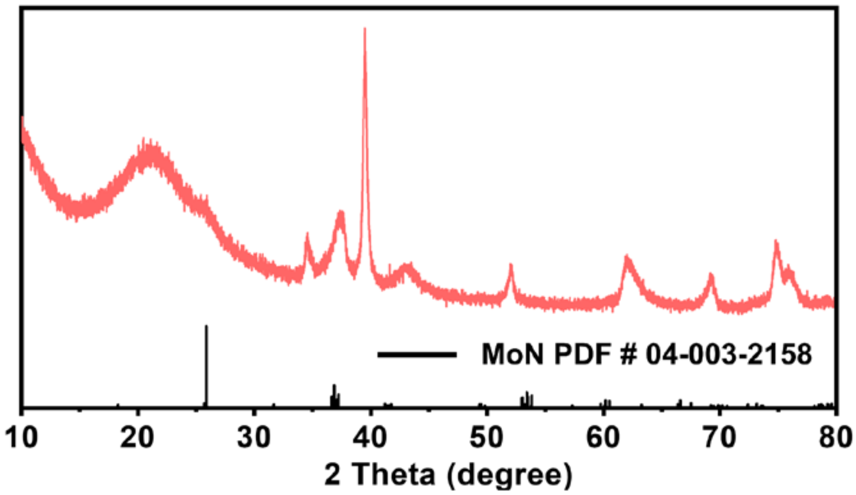

Supplementary Fig. 2 Comparison between the XRD pattern of Mo_2C/N-CNT and the standard PDF card of MoN.

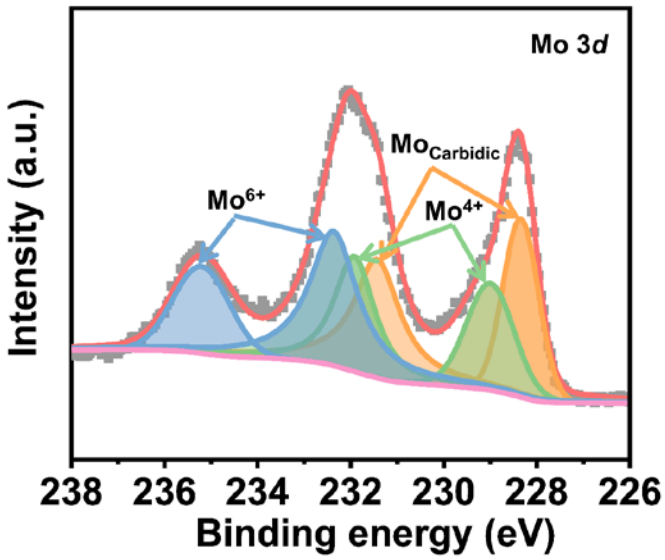

Supplementary Fig. 3 XPS result of Mo 3d of the Mo_2C/N-CNT after laser irradiation in air atmosphere. The a.u. stands for arbitrary units.

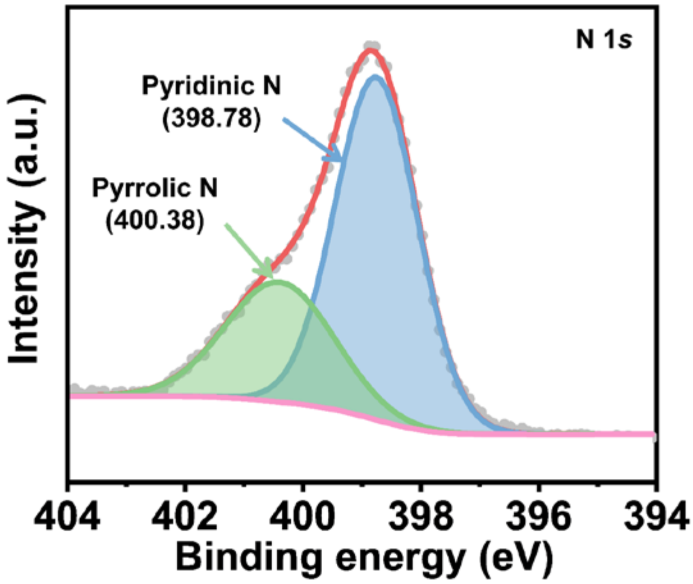

Supplementary Fig. 4 XPS result of N 1*s* of the N-CNT. The a.u. stands for arbitrary units.

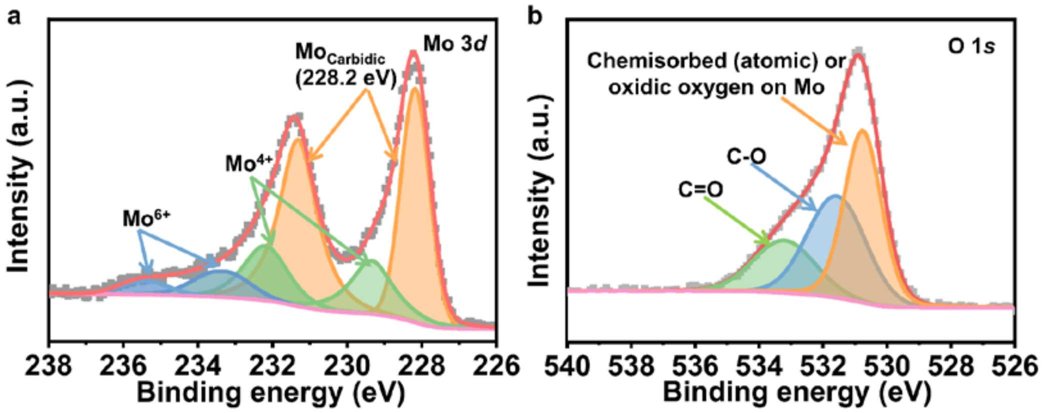

Supplementary Fig. 5 XPS result of Mo_2C/N-CNT before Ar^+ etching. (**a**) XPS result of Mo 3*d* of the Mo_2C/N-CNT before Ar^+ etching. (**b**) XPS result of O 1*s* of the Mo_2C/N-CNT. The a.u. stands for arbitrary units.

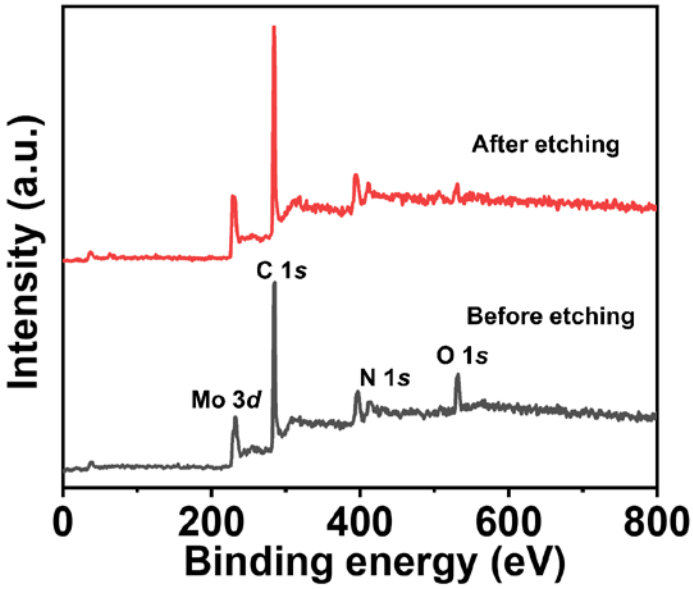

Supplementary Fig. 6 XPS result of Mo_2C/N-CNT before Ar^+ etching and after Ar^+ etching. The a.u. stands for arbitrary units.

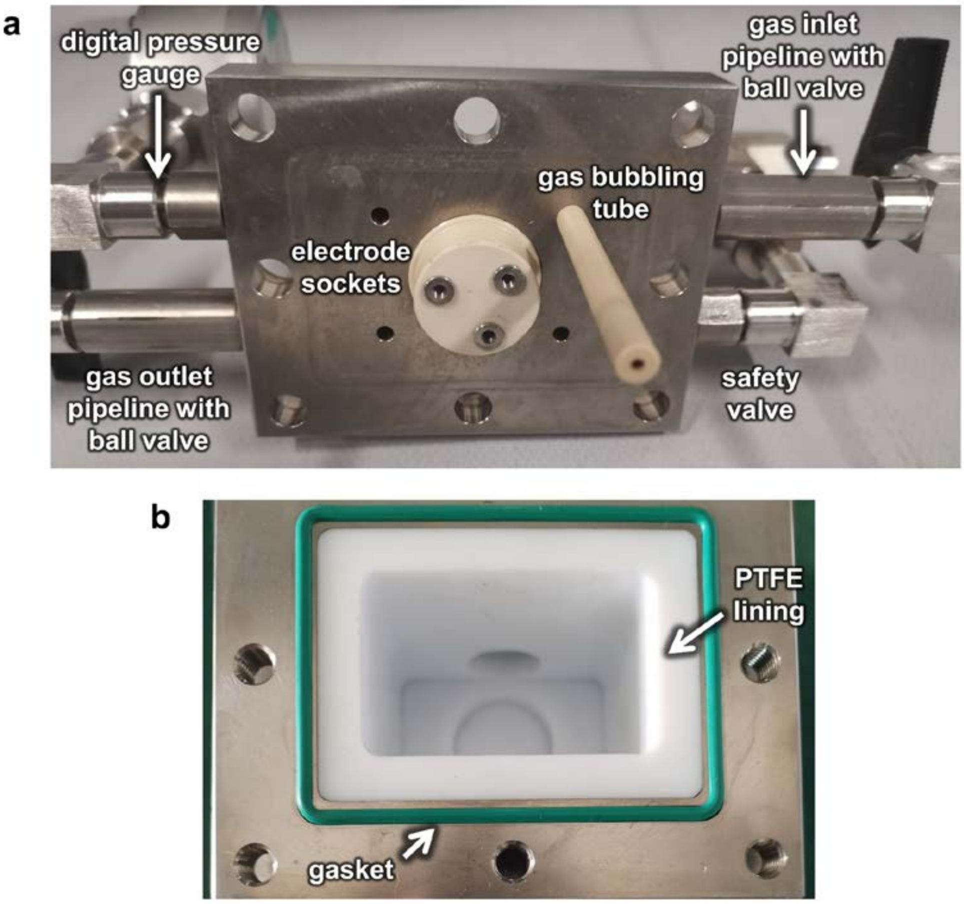

Supplementary Fig. 7 Photograph of high-pressure electrolyser. Photograph of (**a**) chamber cover and (**b**) chamber cell body of high-pressure electrolyser.

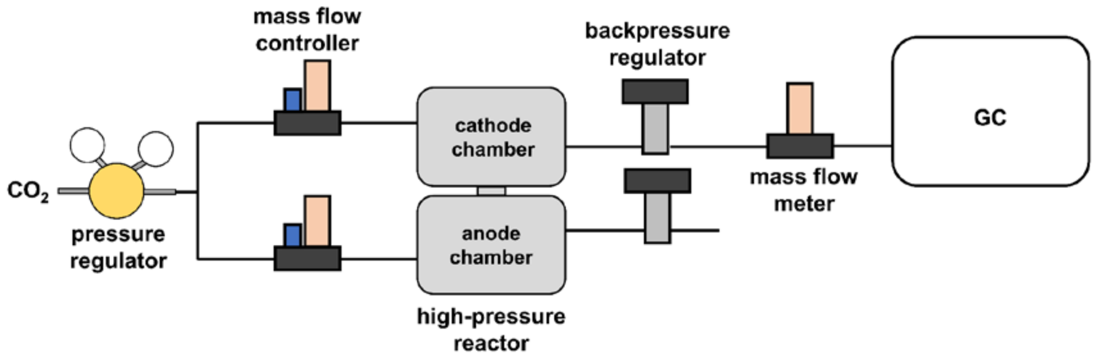

Supplementary Fig. 8 Schematic flowchart of the measurement setup.

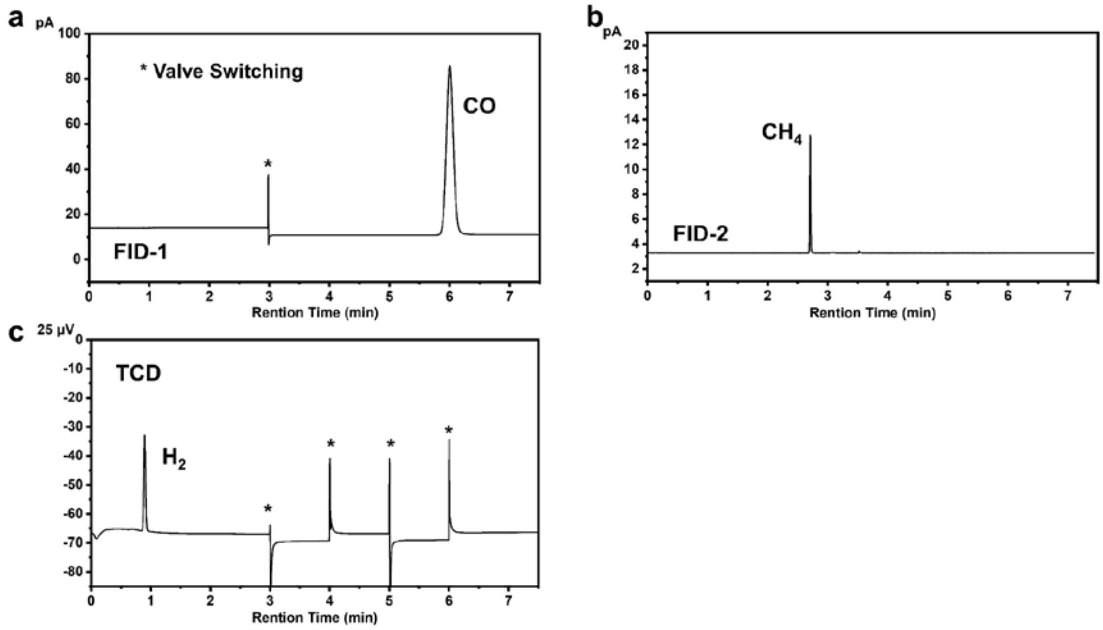

Supplementary Fig. 9 Typical GC diagrams for gas products. (**a**) TCD, (**b**) FID and the (**c**) TCD after 3-hour of CO_2RR catalyzed by Mo_2C/N-CNT at −1.1 V vs. SHE under 40 atm CO_2 pressure conditions.

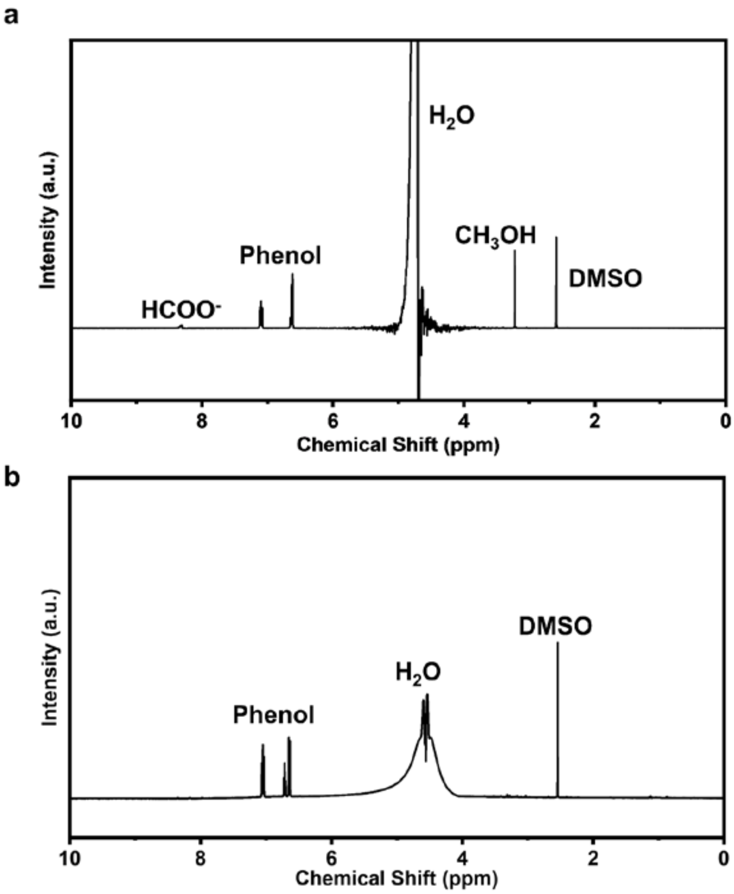

Supplementary Fig. 10 Typical ^{1}H-NMR spectra of a liquid sample. (**a**) catholyte and (**b**) anolyte after 3 h of CO_2RR catalyzed by Mo_2C/N-CNT at −1.1 V vs. SHE under 40 atm CO_2 pressure conditions. The a.u. stands for arbitrary units.

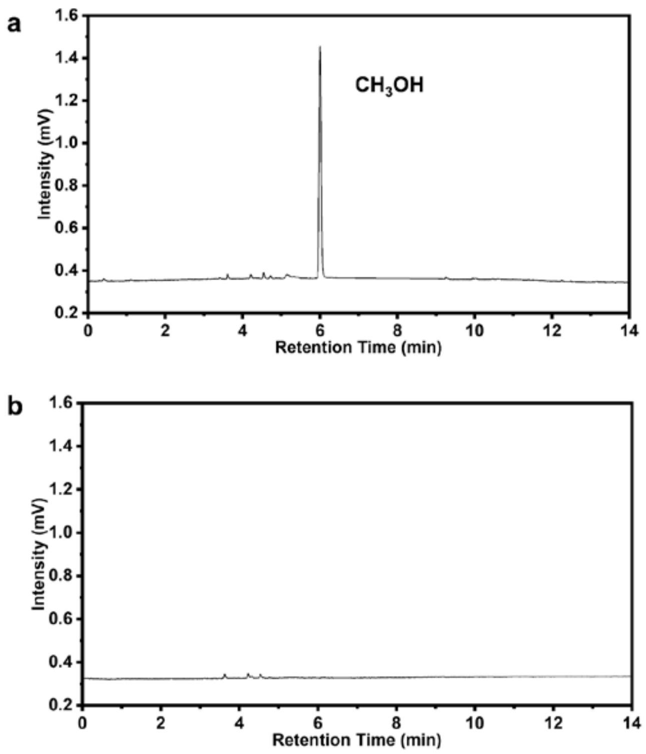

Supplementary Fig. 11 Typical HS-GC diagrams for liquid products detection. (**a**) catholyte and (**b**) anolyte after 3 h of CO_2RR catalyzed by Mo_2C/N-CNT at −1.1 V vs. SHE under 40 atm CO_2 pressure conditions.

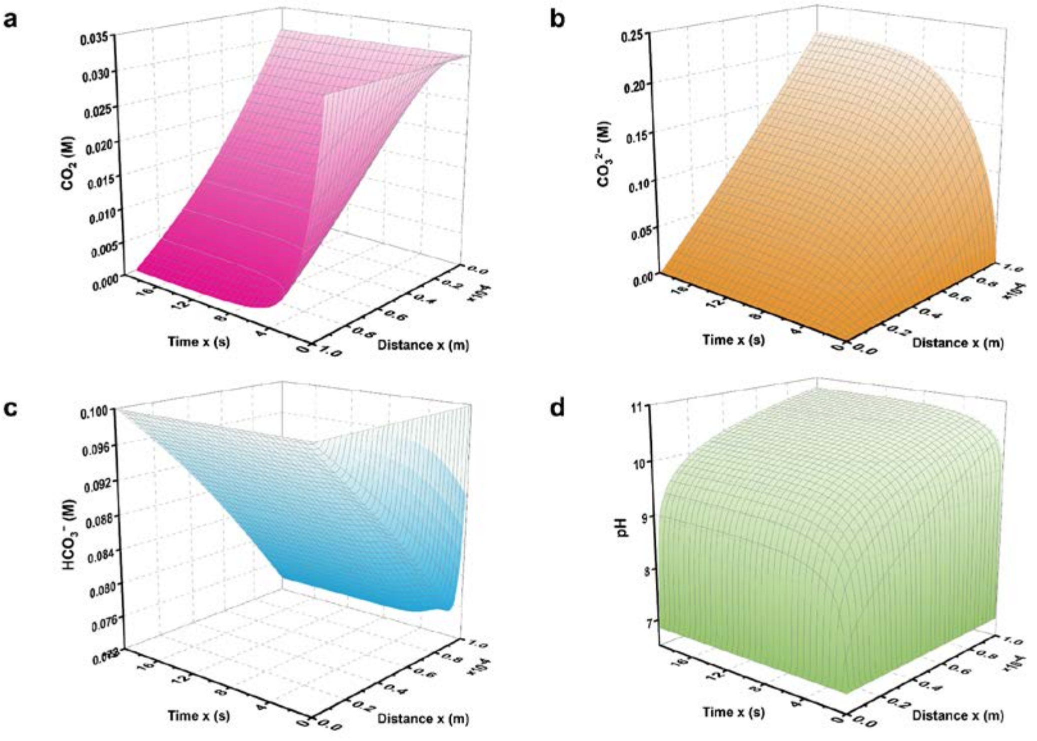

Supplementary Fig. 12 Three-dimensional contours under ambient pressure. (**a**) CO_2, (**b**) CO_3^{2-}, (**c**) HCO_3^- concentrations and (**d**) pH depending on time and location simulated for Mo_2C/N-CNT under ambient pressure.

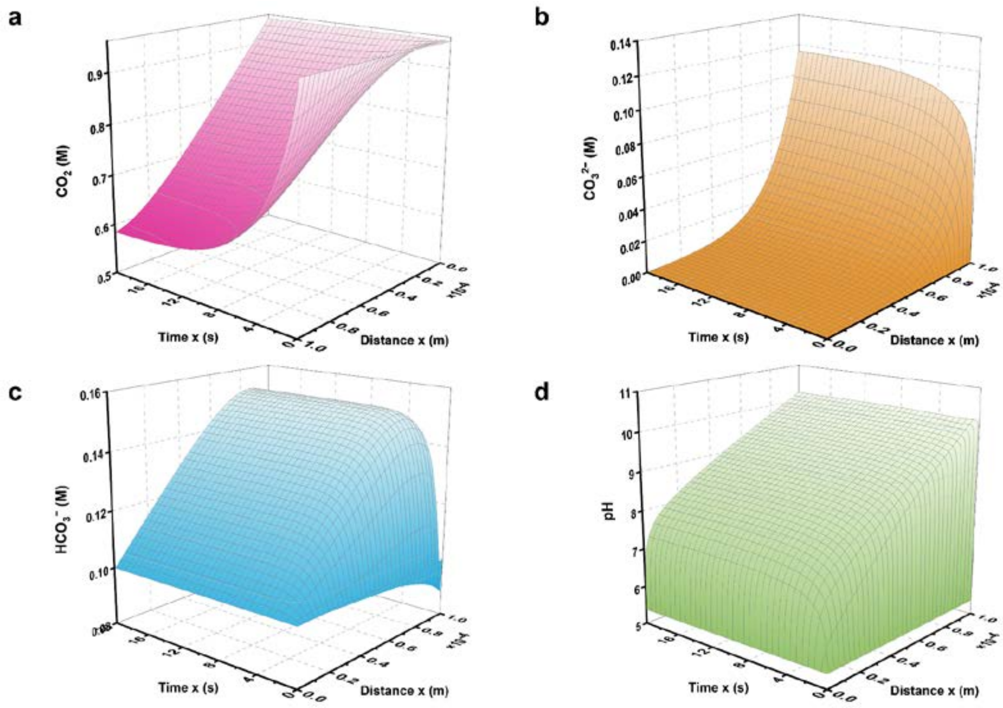

Supplementary Fig. 13 Three-dimensional contours under 4 MPa (40 atm). (**a**) CO_2, (**b**) CO_3^{2-}, (**c**) HCO_3^- concentrations and (**d**) pH depending on time and location simulated for Mo_2C/N-CNT under 4 MPa (40 atm).

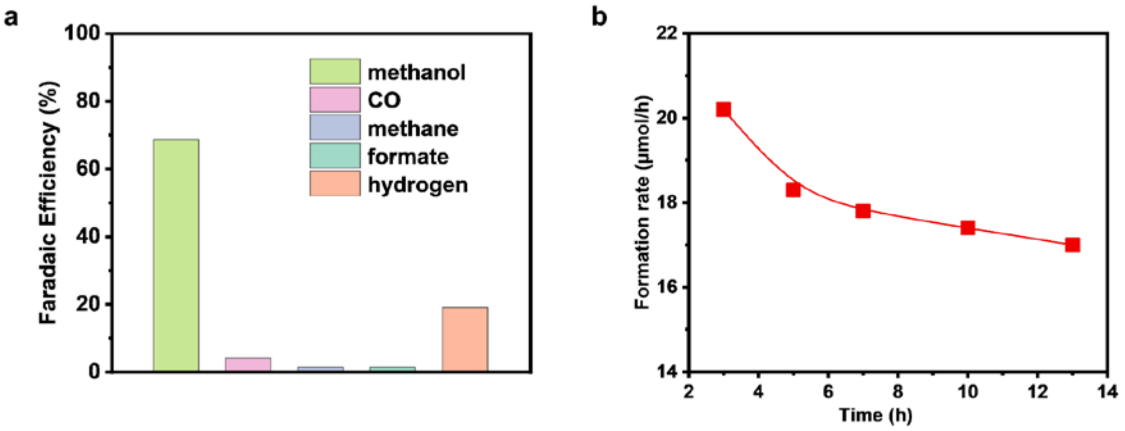

Supplementary Fig. 14 The stability tests of Mo_2C/N-CNT. (**a**) The final product distribution after the stability test. (**b**) Time evolution of CH_3OH formation through CO_2RR catalyzed by Mo_2C/N-CNT at −1.1 V vs. SHE under 40 atm CO_2 pressure conditions.

It is worth noting that the liquid inside the high-pressure reactor cannot be sampled in real-time like the gaseous product. For this reason, the results shown in this figure and the results shown in Fig 2c were obtained from two separate experiments. For the determination of the CH_3OH production rate, multiple batches of CO_2 reduction tests with different reaction durations (each batch using fresh electrolyte) were performed using the same electrode, and the total CH_3OH production after different reaction batches was quantified to calculate the average CH_3OH production rate for the different durations.

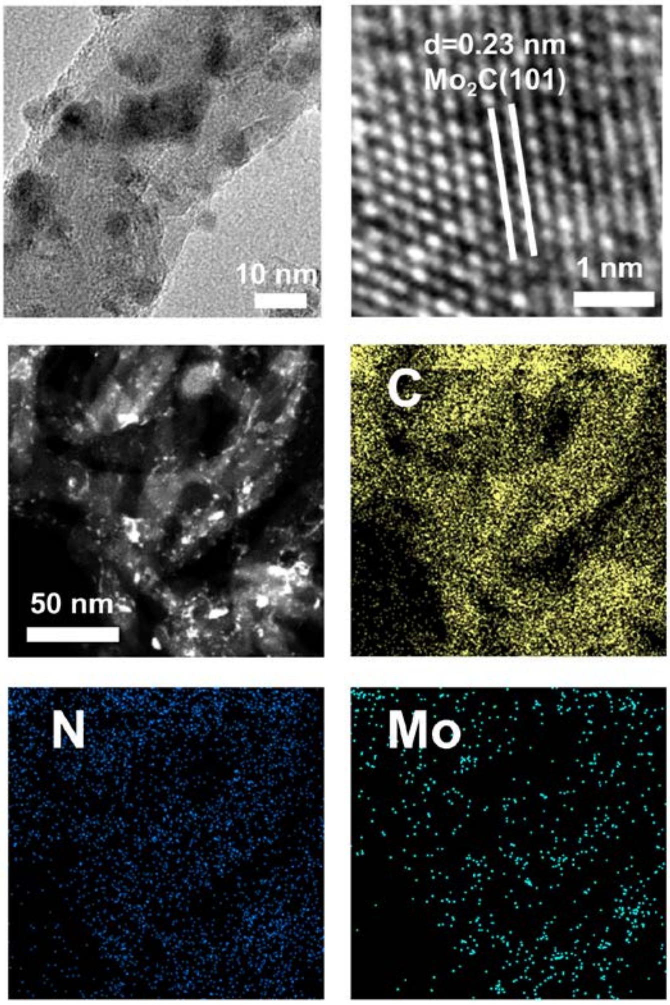

Supplementary Fig. 15 HRTEM and the elemental mapping images of C, N, and Mo of the used Mo_2C/N-CNT.

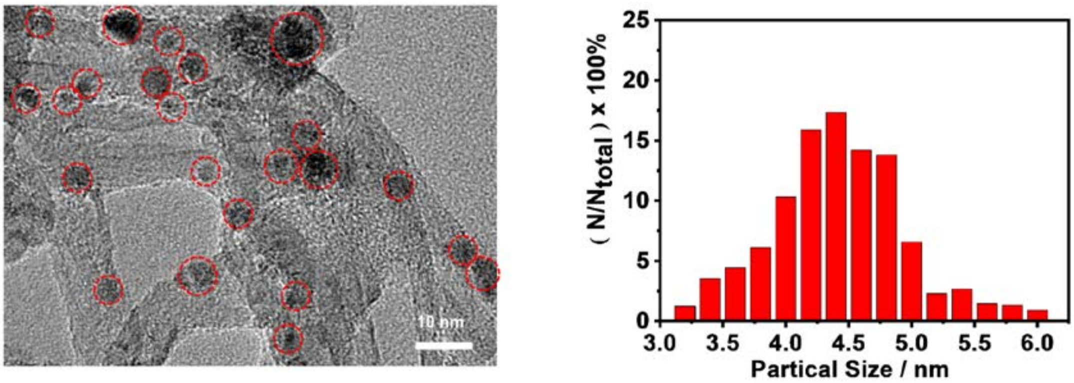

Supplementary Fig. 16 Size distribution of Mo_2C nanoparticles on used Mo_2C/N-CNT.

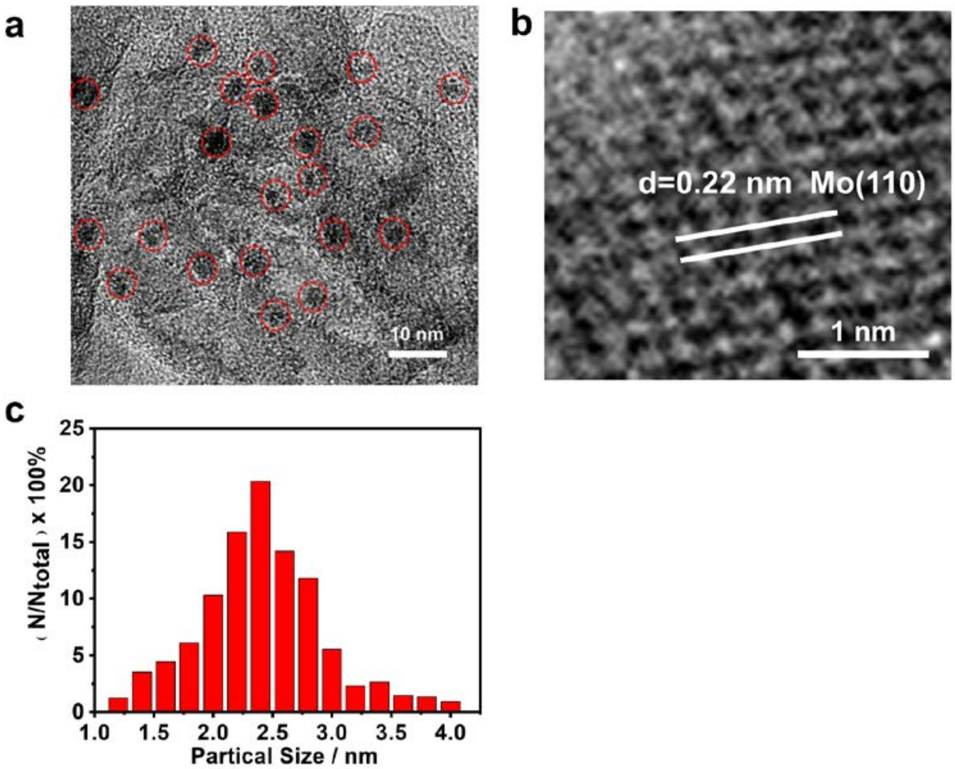

Supplementary Fig. 17 The characterization of the Mo/N-CNT. (**a**) The TEM and (**b**) HRTEM images of the Mo/N-CNT. (**c**) Size distribution of Mo nanoparticles on N-CNT.

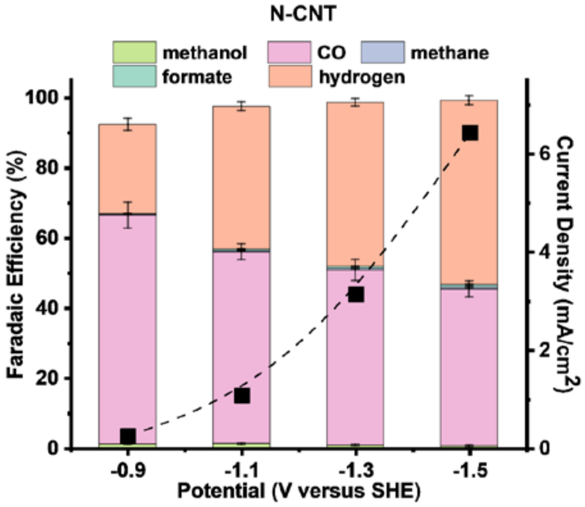

Supplementary Fig. 18 Product distribution of N-CNT. After 3-hour CO_2RR under 40 atm at various potentials. Error bars represent the standard deviation from at least three independent measurements.

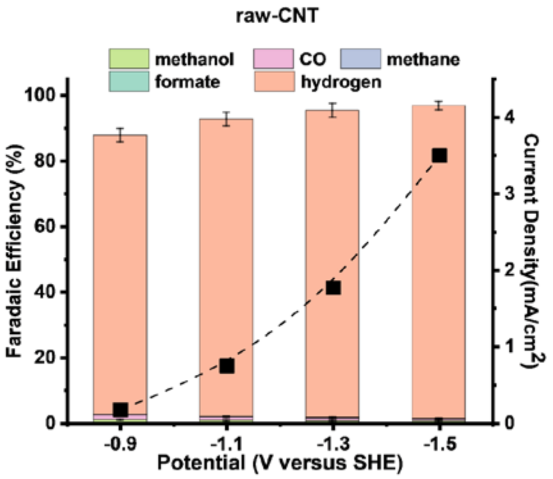

Supplementary Fig. 19 Product distribution of raw-CNT. After 3-hour CO_2RR under 40 atm at various potentials. Error bars represent the standard deviation from at least three independent measurements.

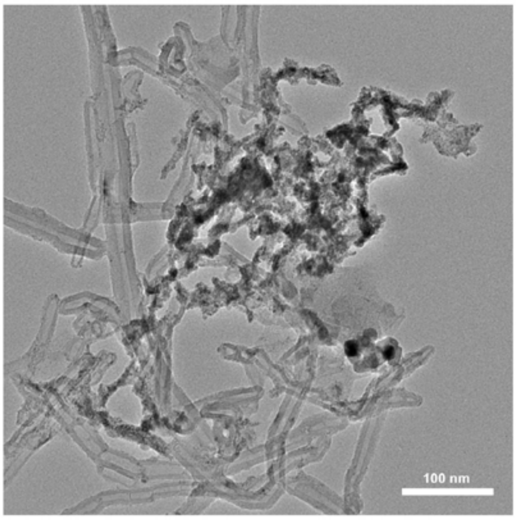

Supplementary Fig. 20 The TEM image of Mo_2C/raw-CNT.

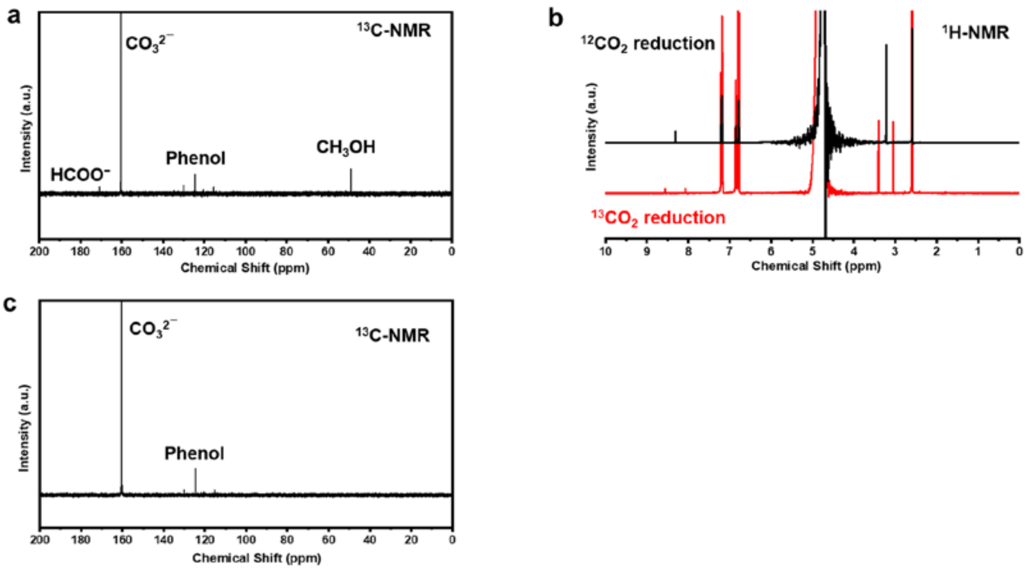

Supplementary Fig. 21 Typical NMR spectra of a liquid sample. (**a**) Typical ^{13}C-NMR and (**b**) ^{1}H-NMR spectra of a liquid sample after $^{13}CO_2$ electroreduction versus (**c**) a blank 0.1 M $KHCO_3$ solution. The a.u. stands for arbitrary units.

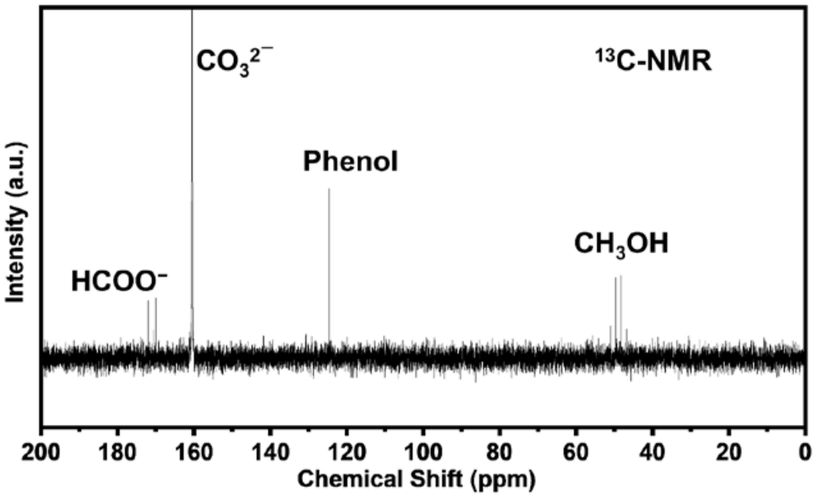

Supplementary Fig. 22 Typical NMR spectra of liquid sample from $^{13}CO_2$ reduction (gated decoupling). The a.u. stands for arbitrary units.

The interaction between ^{13}C and H atoms can be demonstrated using gated decoupling techniques. The $^{1}J_{CH}$ measured experimentally is about 141 Hz.

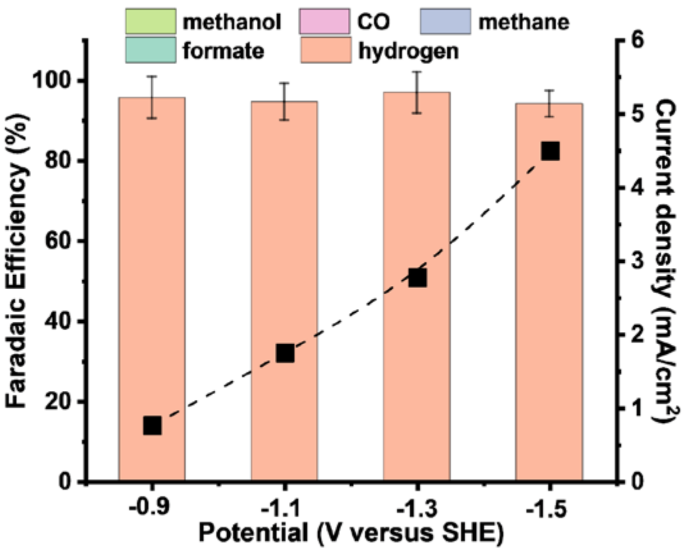

Supplementary Fig. 23 Product distribution of Mo_2C/N-CNT after a 3-hour control test where Ar was introduced as the only gas supply under 40 atm at various potentials. Potassium phosphate buffer adjusted to pH 5.4 was used as electrolyte. Error bars represent the standard deviation from at least three independent measurements.

The carbonaceous product was below the detection limit of the chromatography (Agilent GC7890B) throughout the Ar reduction control test. Therefore, it could be concluded that CH_3OH only originates from the CO_2RR in this study.

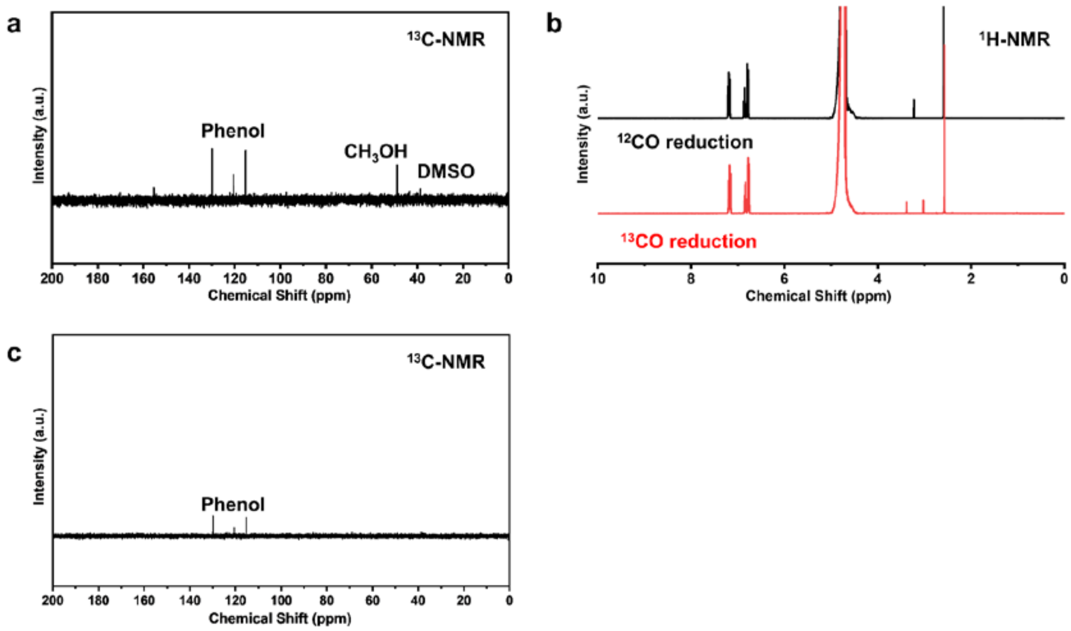

Supplementary Fig. 24 Typical NMR spectra of liquid sample. (**a**) Typical ^{13}C-NMR and (**b**) ^{1}H-NMR spectra of a liquid sample after ^{13}CO electroreduction versus (**c**) a blank potassium phosphate buffer. The a.u. stands for arbitrary units.

Supplementary Fig. 25 Current densities of Mo_2C/raw-CNT during 3-hour CH_2O reduction with 0.1 M potassium phosphate buffer (containing 100 mM CH_3OH-free CH_2O) as electrolyte under 40 atm Ar at various potentials.

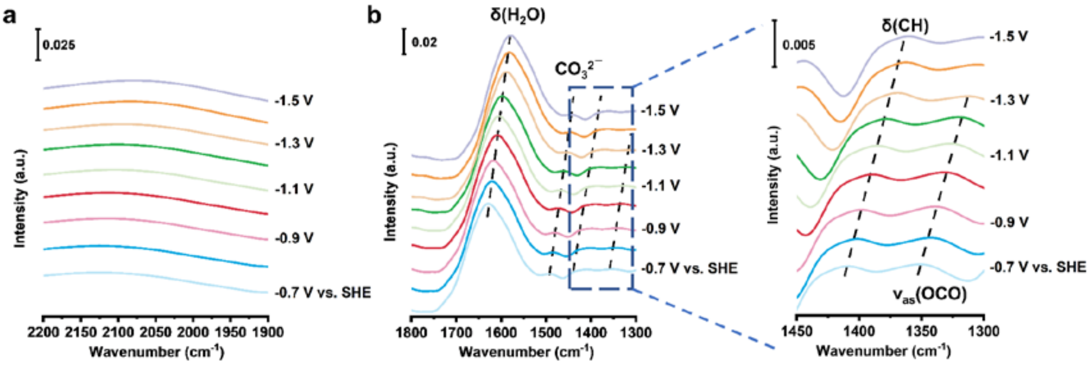

Supplementary Fig. 26 In-situ ATR-SEIRAS spectra of Mo_2C/N-CNT under the ambient pressure. The a.u. stands for arbitrary units.

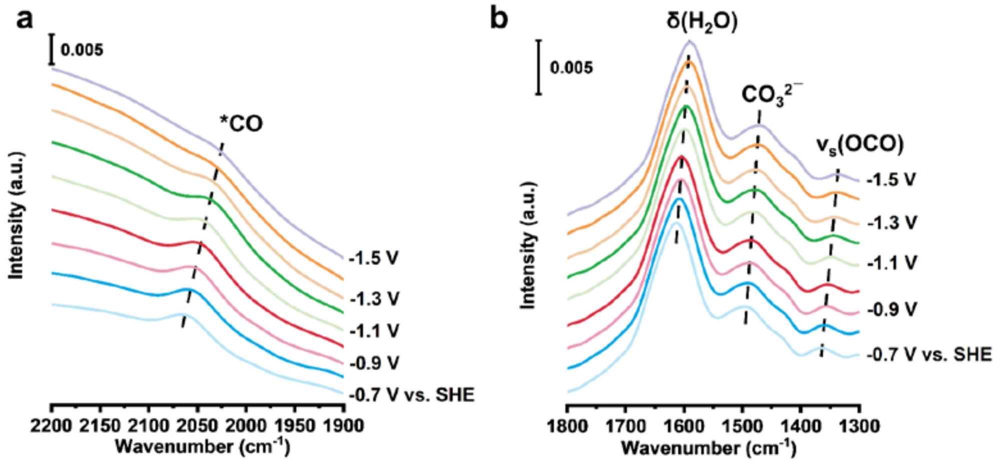

Supplementary Fig. 27 In-situ ATR-SEIRAS spectra of Mo/N-CNT under the ambient pressure. The a.u. stands for arbitrary units.

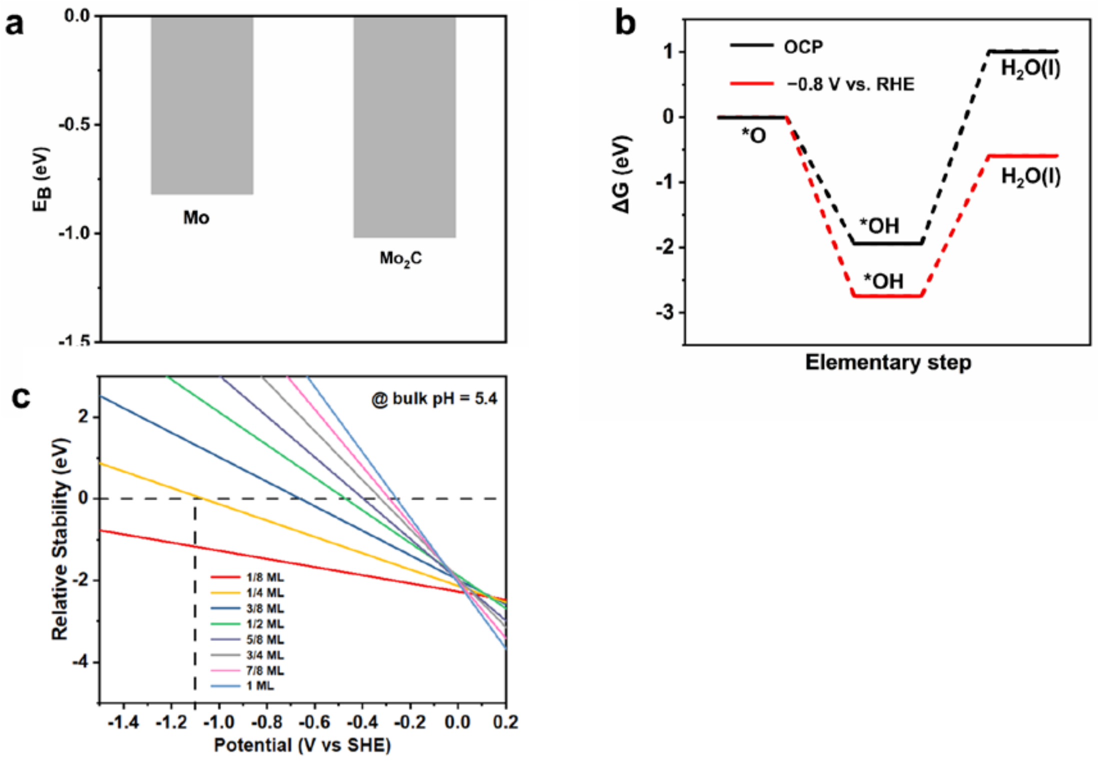

Supplementary Fig. 28 DFT calculations of hydroxyl formation. (**a**) Comparison of the binding energy of O on Mo_2C surfaces versus on its parent Mo metal. (**b**) The Gibbs free energy diagrams of the *O to H_2O on Mo_2C surface at 0 V vs. RHE and –0.8 V vs. RHE. (**c**) Relative stability of hydroxyl-modified Mo_2C(101) with different *OH coverages.

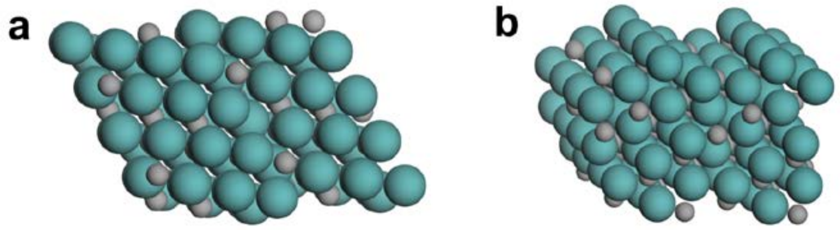

Supplementary Fig. 29 The extended (2×2) Mo_2C slab model. (**a**) The top view. (**b**) The side view. The adsorbed hydroxyl has been removed to show the atoms arrangement. Grey: carbon atoms. Cyan: Molybdenum atoms

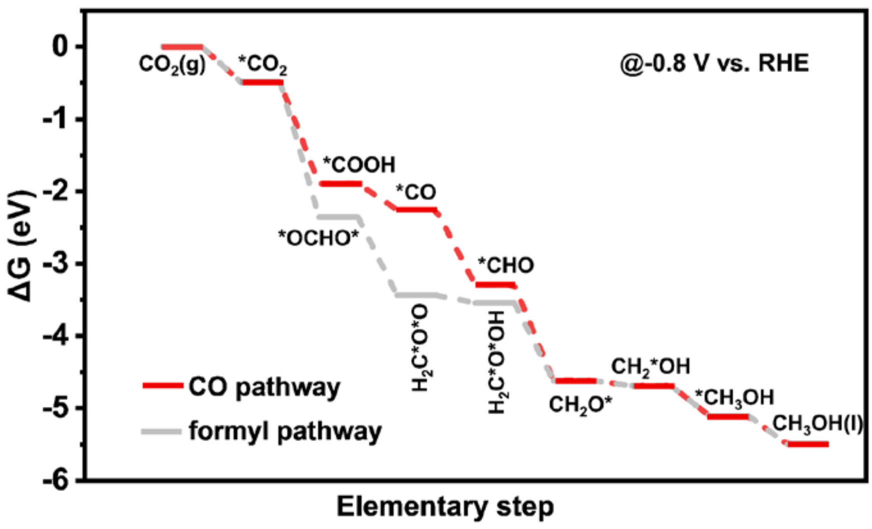

Supplementary Fig. 30 The Gibbs free energy diagrams of the CO_2 to CH_3OH on hydroxyl modified Mo_2C at –0.8 V vs. RHE.

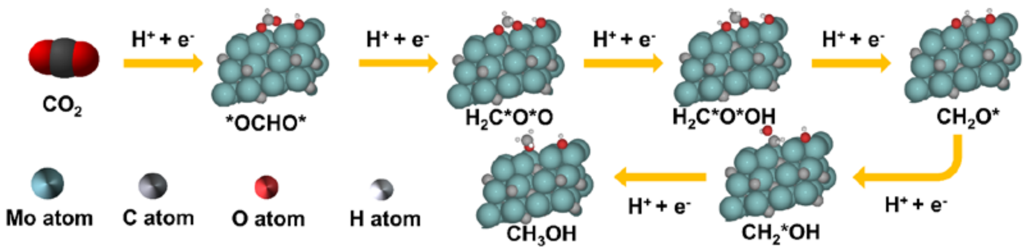

Supplementary Fig. 31 The favorable reaction intermediate configurations on the hydroxyl modified Mo_2C/N-CNT surface (with hydroxyl co-adsorption).

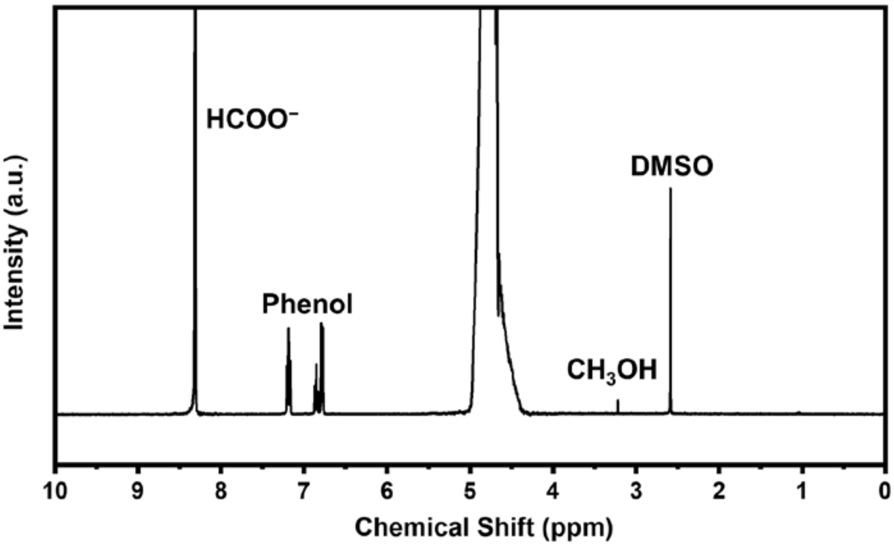

Supplementary Fig. 32 Typical ^{1}H-NMR spectra of a liquid sample after HCOOH electroreduction. The a.u. stands for arbitrary units.

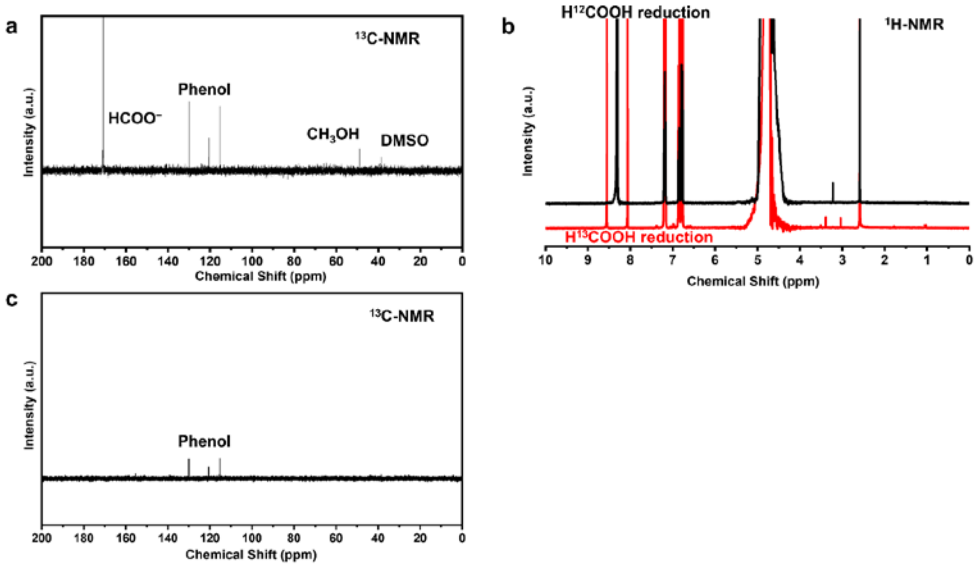

Supplementary Fig. 33 Typical NMR spectra of liquid sample. (**a**) Typical ^{13}C-NMR and (**b**) ^{1}H-NMR spectra of a liquid sample after H^{13}COOH electroreduction versus (**c**) a blank potassium phosphate buffer. The a.u. stands for arbitrary units.

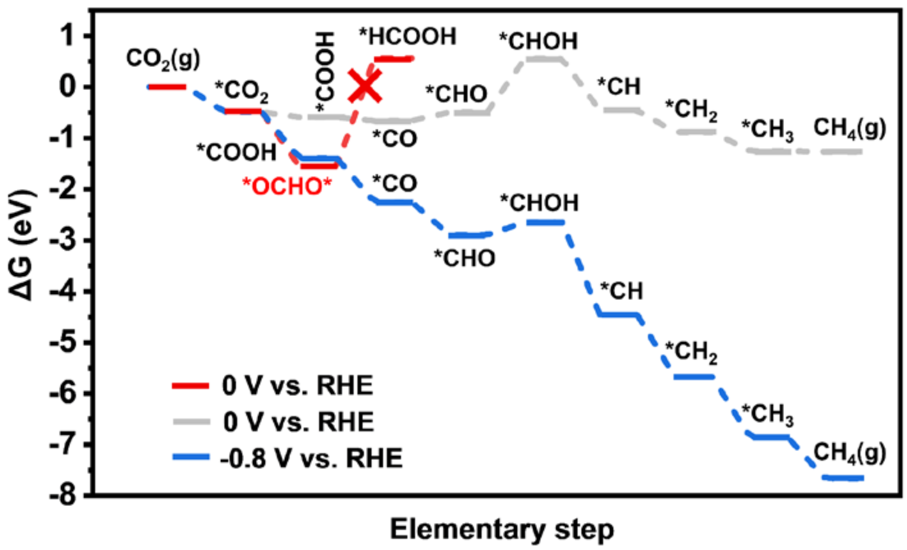

Supplementary Fig. 34 The Gibbs free energy diagrams of the CO_2 to HCOOH and CH_4 on hydroxyl modified Mo_2C at 0 V vs. RHE and –0.8 V vs. RHE.

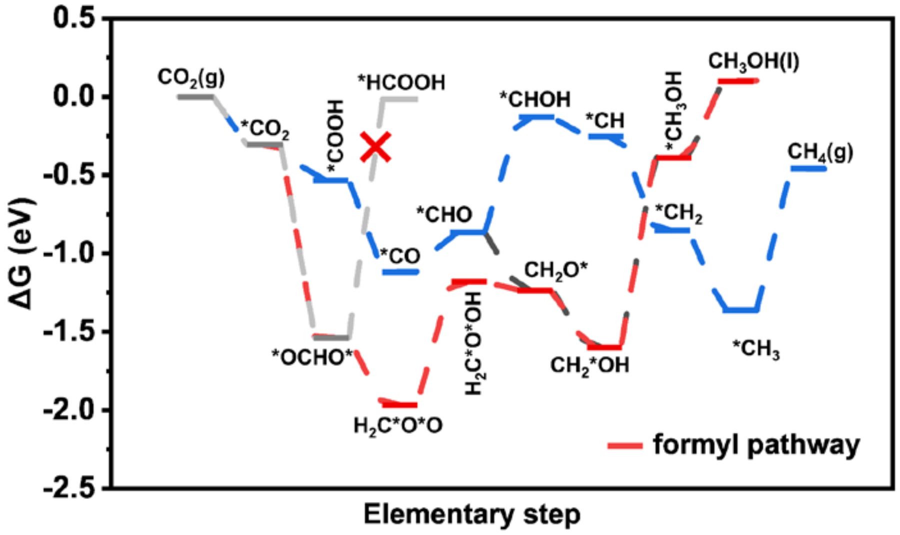

Supplementary Fig. 35 The Gibbs free energy diagrams of the CO_2 to CH_3OH, HCOOH and CH_4 on bare Mo_2C at 0 V vs. RHE.

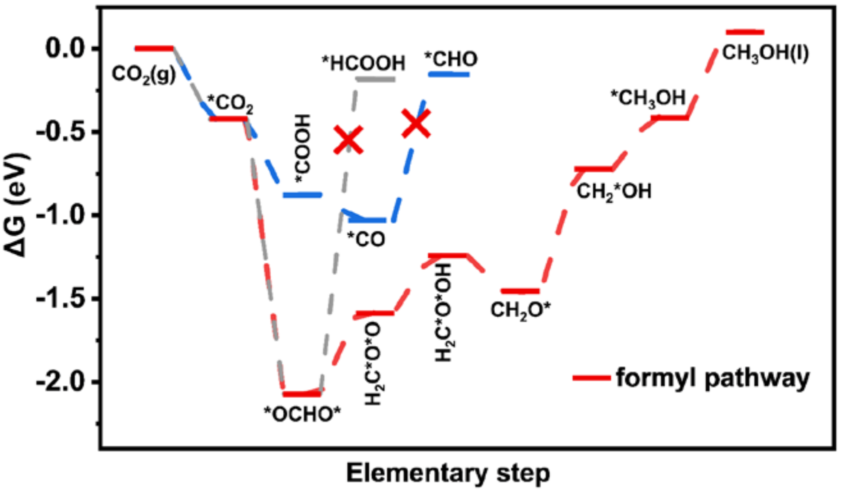

Supplementary Fig. 36 The Gibbs free energy diagrams of CO_2 reduction on bare Mo at 0 V vs. RHE.

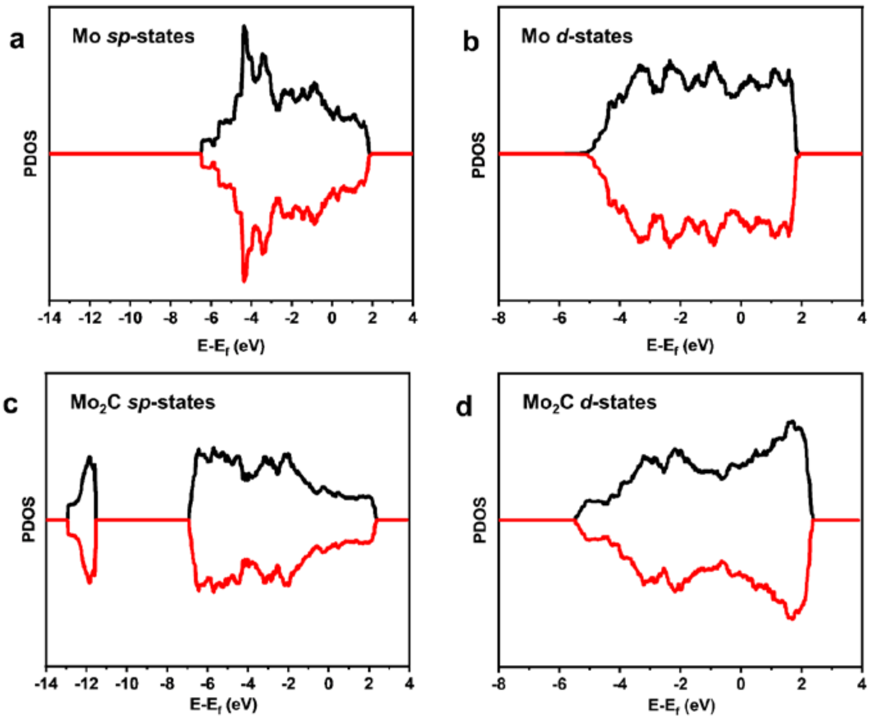

Supplementary Fig. 37 Representative Mo-projected DOS of various samples. (**a, b**) Mo(110) and (**c d**) Mo_2C(101).

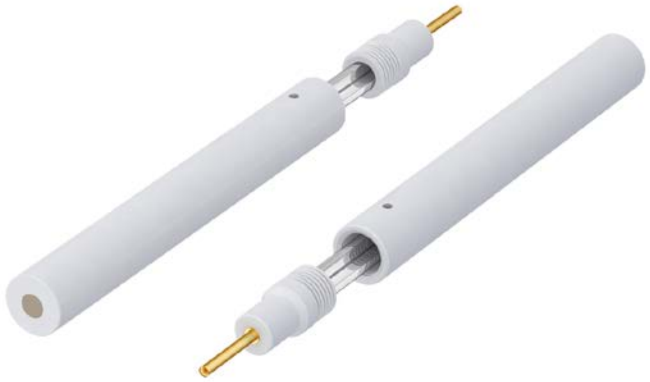

Supplementary Fig. 38 Schematic of the self-designed saturated Ag/AgCl electrode with a salt bridge.

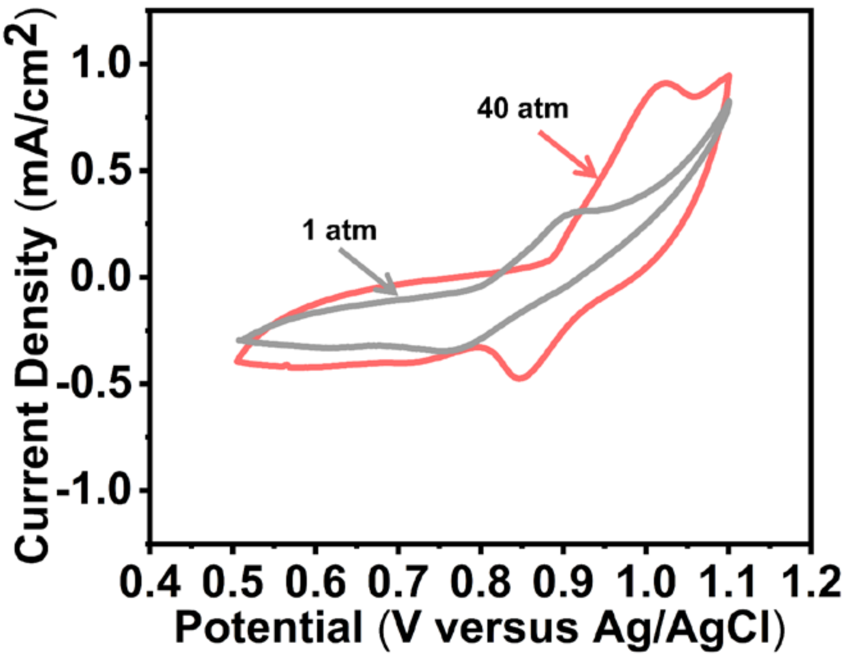

Supplementary Fig. 39 The cyclic voltammetry curve of NiFe-hydroxide carbonate. The curve was obtained in 0.1 M $KHCO_3$ under 40 atm CO_2 (the corresponding electrolyte pH is 5.4), with a scan rate of 5 mV/s.[6]

According to the Nernst equation, the peak potential should be shifted by 87 mV at a CO_2 pressure of 40 atm (the electrolyte pH is 5.4), compared to that at a CO_2 pressure of 1 atm (the electrolyte pH is 6.8).

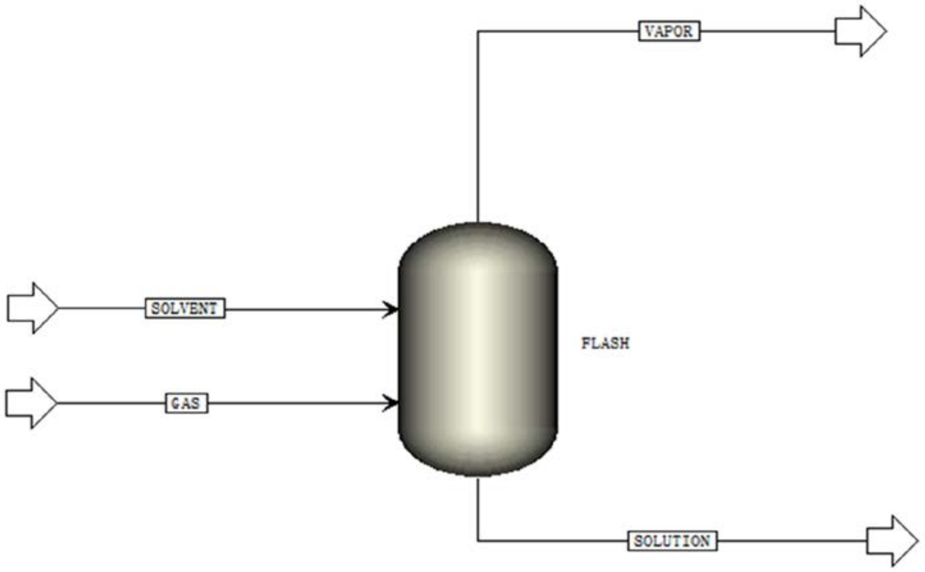

Supplementary Fig. 40 Illustration of the Aspen simulation model used for the calculation of CO_2 solubility.

The obtained CO_2 solubility results were listed in Supplementary Table 12.

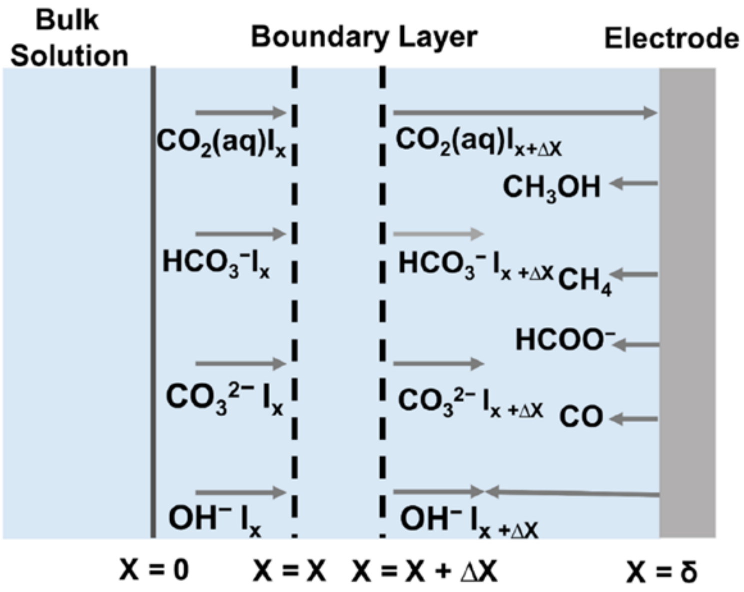

Supplementary Fig. 41 Illustration of the reaction-mass transport model for CO_2RR.

Supplementary Table 1 Summary of representative CO_2RR results on CH_3OH formation.

Catalysts	Potential (V vs. RHE)	Current Density (mA/cm^2)	CH_3OH FE	Ref.
Co-Phthalocyanine	−0.74	0.680	19.5%	7
Pd/SnO_2	−0.24	0.794	54.8%	8
BP	−0.5	8.00×10^{-2}	92%	9
Cu_2O/polypyrrole	−0.85	0.223	93%	10
Ag-Zn	−1.39	2.70	10.5%	11
Cu_2O/MWCNTs	−0.17	2.28	38%	12
Co/N-Graphene	−0.25	2.86	71.4%	13
FeP	−0.9	1.50	80.2%	14
Mo_2C/M-CNT	−0.8 (−1.1 V vs. SHE)	4.20	80.4%	This Work

Supplementary Table 2. The free energy of *CO desorption and *CO to *CHO on different slabs. All values are given in eV.

Slab	*CO desorption	*CO →*CHO
hydroxyl modified-Mo_2C(101)	1.28	–0.24
bare Mo_2C(101)	1.74	0.25
Mo(110)	1.65	0.88

Supplementary Table 3 Calculated adsorbed *OCHO* frequency (cm^{-1}) on hydroxyl modified Mo_2C surface and *COOH frequency (cm^{-1}) on bare Mo.

Calculations		Experiments	
Slab	ν/ cm^{-1}	Sample	ν/ cm^{-1}
hydroxyl modified-Mo_2C(101)	1371	Mo_2C(101)	1367
bare Mo(110)	1362	Mo(110)	1355

Supplementary Table 4 The calculated HCOOH adsorption energy and the free energy of HCOOH hydrogenation reaction on hydroxyl modified-Mo_2C(101). All values are given in eV.

Reaction step	Free energy
*CO adsorption	−1.28
*HCOOH adsorption	0.41
HCOOH to H_2C*O*OH	0.39

Supplementary Table 5. Charge transfer from the different slabs to the key adsorbates. All values are given in e.

Slab	*CO	*CHO	CH_2O*
hydroxyl modified-Mo_2C(101)	0.39	0.58	0.70
bare Mo_2C(101)	0.50	0.90	0.79

Supplementary Table 6 The coordination of each atom in optimized structures

Coordination (cartesian) of the hydroxyl modified Mo_2C

Unit cell:

	x/Å	y/Å	z/Å
x	7.68	0.00	0.00
y	–4.79	11.15	0.00
z	0.00	0.00	25.27

Atom	x/Å	y/Å	z/Å
C	2.76	2.38	7.76
C	–0.11	1.14	11.27
C	–0.14	11.02	14.93
Mo	3.22	0.98	9.27
Mo	–4.50	10.94	12.85
Mo	2.92	3.86	16.06
Mo	3.46	0.97	6.30
Mo	–4.22	10.88	9.81
Mo	2.96	4.13	13.31
C	0.37	3.00	6.00
C	5.16	1.76	9.51
C	2.28	0.51	13.02
Mo	0.82	1.60	7.51
Mo	5.62	0.36	11.02
Mo	0.36	4.60	14.42
Mo	5.86	0.35	8.05
Mo	0.60	4.70	11.57
Mo	5.28	3.41	14.98
C	5.41	5.17	7.76
C	2.52	3.93	11.26
C	–0.34	2.71	15.00
Mo	–0.33	4.41	7.46
Mo	4.46	3.17	10.96
Mo	1.63	1.96	14.44
Mo	4.71	3.15	8.00
Mo	1.82	1.93	11.55
Mo	6.61	0.77	14.85
C	5.41	0.21	6.00
C	0.12	4.55	9.51
C	4.91	3.31	13.00
Mo	2.07	3.79	9.21
Mo	–0.81	2.52	12.73

Mo	3.71	1.39	15.96
Mo	2.31	3.77	6.25
Mo	–0.57	2.53	9.75
Mo	4.27	1.28	13.26
C	0.37	7.95	7.76
C	–2.51	6.72	11.27
C	2.26	5.45	14.92
Mo	0.82	6.56	9.27
Mo	–2.10	5.36	12.85
Mo	0.49	9.33	15.95
Mo	1.07	6.54	6.30
Mo	–1.82	5.30	9.81
Mo	0.56	9.71	13.33
C	–2.03	8.57	6.00
C	2.77	7.33	9.51
C	–0.12	6.09	13.03
Mo	–1.57	7.18	7.51
Mo	3.22	5.94	11.02
Mo	–2.06	10.21	14.45
Mo	3.46	5.92	8.05
Mo	–1.80	10.27	11.56
Mo	2.92	8.97	14.99
C	3.01	10.74	7.76
C	0.12	9.49	11.27
C	–2.74	8.28	14.99
Mo	–2.73	9.99	7.46
Mo	2.07	8.75	10.96
Mo	–0.77	7.55	14.49
Mo	2.31	8.73	8.00
Mo	–0.57	7.49	11.53
Mo	4.23	6.30	14.86
C	3.01	5.79	6.00
C	–2.27	10.12	9.51
C	2.52	8.90	12.99
Mo	–0.33	9.37	9.21
Mo	–3.20	8.10	12.74
Mo	1.40	7.05	15.93
Mo	–0.09	9.35	6.25
Mo	–2.97	8.11	9.75
Mo	1.86	6.87	13.30
O	3.24	2.52	17.66
H	3.80	2.66	18.44

Coordination (cartesian) of the HCOO* on hydroxyl modified Mo_2C

Unit cell:

	x/Å	y/Å	z/Å
x	7.68	0.00	0.00
y	–4.79	11.15	0.00
z	0.00	0.00	25.27

Atom	x/Å	y/Å	z/Å
C	2.76	2.38	6.96
C	–0.11	1.14	10.47
C	–0.12	10.99	14.13
Mo	3.22	0.98	8.47
Mo	–4.50	10.93	12.05
Mo	2.96	3.89	15.24
Mo	3.46	0.97	5.50
Mo	–4.22	10.88	9.01
Mo	2.96	4.14	12.52
C	0.37	3.00	5.20
C	5.16	1.76	8.71
C	2.29	0.50	12.22
Mo	0.82	1.60	6.71
Mo	5.62	0.36	10.22
Mo	0.36	4.62	13.63
Mo	5.86	0.35	7.25
Mo	0.60	4.70	10.77
Mo	5.28	3.42	14.17
C	5.41	5.17	6.96
C	2.52	3.93	10.46
C	–0.33	2.71	14.18
Mo	–0.33	4.41	6.66
Mo	4.46	3.17	10.16
Mo	1.65	1.97	13.64
Mo	4.71	3.15	7.20
Mo	1.82	1.92	10.75
Mo	6.61	0.76	14.04
C	5.41	0.21	5.20
C	0.12	4.55	8.71
C	4.92	3.31	12.19
Mo	2.07	3.79	8.41
Mo	–0.81	2.52	11.93
Mo	3.70	1.37	15.14
Mo	2.31	3.77	5.45

Mo	–0.57	2.53	8.95
Mo	4.28	1.27	12.47
C	0.37	7.95	6.96
C	–2.51	6.72	10.47
C	2.25	5.49	14.10
Mo	0.82	6.56	8.47
Mo	–2.09	5.36	12.04
Mo	0.51	9.39	15.29
Mo	1.07	6.54	5.50
Mo	–1.82	5.30	9.01
Mo	0.56	9.72	12.49
C	–2.03	8.57	5.20
C	2.77	7.33	8.71
C	–0.11	6.10	12.24
Mo	–1.57	7.18	6.71
Mo	3.22	5.94	10.22
Mo	–2.04	10.20	13.65
Mo	3.46	5.92	7.25
Mo	–1.80	10.27	10.77
Mo	2.88	8.94	14.21
C	3.01	10.74	6.96
C	0.12	9.50	10.46
C	–2.73	8.29	14.18
Mo	–2.73	9.99	6.66
Mo	2.07	8.75	10.16
Mo	–0.73	7.56	13.70
Mo	2.31	8.73	7.20
Mo	–0.57	7.50	10.74
Mo	4.23	6.32	14.05
C	3.01	5.79	5.20
C	–2.27	10.12	8.71
C	2.52	8.89	12.21
Mo	–0.33	9.37	8.41
Mo	–3.20	8.11	11.94
Mo	1.40	6.98	15.22
Mo	–0.09	9.35	5.45
Mo	–2.97	8.11	8.95
Mo	1.88	6.85	12.43
O	0.51	9.16	17.37
C	0.78	8.05	17.94
O	1.12	7.00	17.30
H	0.71	7.99	19.04
O	3.24	2.52	16.83
H	3.83	2.67	17.59

Supplementary Table 7 The zero-point energy correction, enthalpy correction and entropy correction for adsorbates and free molecules. All values are given in eV.

Types	Adsorbates	ZPE	δH_0	TS
bare Mo_2C	*COOH	0.60	0.11	0.22
	OCHO	0.62	0.10	0.19
	*CO	0.18	0.08	0.15
	*HCOOH	0.89	0.12	0.24
	H_2C*O*O	0.91	0.09	0.19
	H_2C*O*OH	0.92	0.10	0.20
	*CHO	0.47	0.05	0.09
	CH_2O*	0.68	0.07	0.21
	CH_2*OH	1.21	0.13	0.31
	*CH_3OH	1.39	0.13	0.33
	*CHOH	0.74	0.10	0.12
	*CH	0.33	0.04	0.05
	*CH_2	0.58	0.07	0.12
	*CH_3	0.89	0.09	0.13
	*CO_2	0.99	0.17	0.33
	*OH	0.35	0.05	0.09
hydroxyl modified Mo_2C	*COOH	0.61	0.10	0.22
	OCHO	0.63	0.10	0.19
	*CO	0.18	0.07	0.15
	*HCOOH	0.90	0.12	0.23
	H_2C*O*OH	0.91	0.10	0.20
	H_2C*O*O	1.21	0.13	0.31
	*CHO	0.46	0.07	0.13
	CH_2O*	0.72	0.14	0.44
	CH_2*OH	1.01	0.11	0.24
	*CH_3OH	1.40	0.13	0.33
	*CHOH	0.76	0.08	0.14
	*CH	0.33	0.04	0.07
	*CH_2	0.70	0.04	0.06
	*CH_3	0.90	0.07	0.13
	*CO_2	0.25	0.11	0.21
bare Mo	*COOH	0.61	0.10	0.21
	OCHO	0.63	0.09	0.18
	*CO	0.18	0.07	0.18
	H_2C*O*O	0.88	0.09	0.16
	*HCOOH	0.90	0.12	0.22
	*H_2C*O*OH	0.89	0.18	0.25
	*CHO	0.40	0.07	0.20
	CH_2O*	0.74	0.07	0.12
	CH_2*OH	0.73	0.09	0.11

Others	CO_2	0.31	0.11	0.66
	CO	0.14	0.10	0.76
	H_2O	0.58	0.10	0.65
	H_2	0.28	0.09	0.43
	CH_3OH	1.35	0.16	0.59
	CH_4	1.36	0.11	0.71

Supplementary Table 8 The solvent correction for adsorbates. All values are given in eV.

Adsorbates	Solvent correction
*COOH	0.48
OCHO	0.10
*CO	0.10
*HCOOH	0.38
H_2C*O*O	0.38
H_2C*O*OH	0.38
*CHO	0.10
CH_2O*	0.00
CH_2*OH	0.50
*CH_3OH	0.50
*CHOH	0.50
*CH	0.00
*CH_2	0.00
*CH_3	0.00
*OH	0.50

Supplementary Table 9 Reaction rate constants.

k_{1f}	2.23×10^{3} M^{-1} s^{-1}
k_{1r}	5.02×10^{-5} s^{-1}
k_{2f}	6×10^{9} M^{-1} s^{-1}
k_{2r}	1.29×10^{6} s^{-1}

Since the value of the reaction rate constant is only related to temperature, the reaction rate constants under different pressures are approximately equal.[15,16]

Supplementary Table 10 Diffusion coefficients (25 °C, 0.1 M $KHCO_3$) for CO_2, HCO_3^-, CO_3^{2-} and OH^- at different pressures.

	0.1 MPa ($m^2\ s^{-1}$)	4 MPa ($m^2\ s^{-1}$) *
CO_2	1.91×10^{-9}	1.15×10^{-9}
HCO_3^-	9.23×10^{-10}	5.54×10^{-10}
CO_3^{2-}	1.19×10^{-9}	7.14×10^{-10}
OH^-	5.27×10^{-9}	3.16×10^{-9}

* These data were obtained by molecular dynamics simulations.

Supplementary Table 11 Chemical equation of CO_2 reduction.

	CO_2 stoichiometry	OH^- stoichiometry	Electron transfer number N
$CO_2 \leftrightarrow CO + 1/2O_2$	1	2	2
CO_2 + H2O ↔ HCOOH + $1/2O_2$	1	2	2
$CO_2 + 2H_2O \leftrightarrow CH_4 + 2O_2$	1	8	8
$CO_2 + 2H_2O \leftrightarrow CH_3OH + 3/2O_2$	1	6	6
$H_2O \leftrightarrow H_2 + 1/2O_2$	N.A.	2	2
$HCOOH + OH^- \leftrightarrow HCOO^- + H_2O$	N.A.	1	N.A.

Supplementary Table 12 Initial equilibrium values (at t=0) for CO_2, HCO_3^-, CO_3^{2-}, OH^- and bulk pH at different pressures (25 °C).

	0.1 MPa	4 MPa
CO_2	0.03143 M	0.9521 M
HCO_3^-	0.09993 M	0.1000 M
CO_3^{2-}	0.00003450 M	0.000001140 M
OH^-	0.00000007132 M	0.000000002356 M
pH	6.853	5.372

Supplementary Text

1. Assembly method of the high-pressure reactor.

The reactor is divided into two chambers using a bipolar membrane (~160 μm, Fumasep FBM-PK, Fumatech). The headspaces of the two chambers are not connected. Each chamber contains a cover plate and a cell body. The cell body is made of titanium metal with a polytetrafluoroethylene (PTFE) lining wrapped inside to hold the electrolyte (Supplementary Fig. 7). The chamber cover (Supplementary Fig. 7) is mounted with an inlet gas pipeline (316L, with a ball valve, Beijing Xiongchuan Technology Co., Ltd.), an outlet gas pipeline (316L, with a ball valve, Beijing Xiongchuan Technology Co., Ltd.), a gas bubbling tube (poly(ether-ether-ketone), PEEK), a digital pressure gauge (ACD-200mini, Xi'an ANCN Smart Instrument Inc.) and a safety valve (Beijing Xiongchuan Technology Co., Ltd.). The gas bubbling tube will be inserted into the electrolyte. The inlet gas pipeline of each chamber cover is connected downstream to the own gas bubbling tube and upstream to a digital mass flow controller (CS100D, Beijing Sevenstar Flow Co., Ltd.). Meanwhile, the upstream pipelines of the two flow controllers are connected to the CO_2 gas supply through a 316L three-way manifold (Beijing Xiongchuan Technology Co., Ltd.), so that CO_2 can be delivered to both chambers at the same time, avoiding the pressure difference over the bipolar membrane (Supplementary Fig. 8). The outlet gas pipeline of each chamber cover is connected to the respective back pressure regulator (TESCOM, Emerson). The backpressure regulator of the cathode is connected to a digital mass flow meter (M-Series, Alicat Scientific), which is further connected to the online GC (Agilent GC7890B). The backpressure regulator of the anode is directly connected to the atmosphere. This design avoids gas mixing between the cathode and anode. The absence of O_2 in the cathode product confirms the effectiveness of our design (Supplementary Fig. 9). At the backpressure regulator, there exists a drop in gas pressure, which may result in a decrease in gas temperature according to the Joule Thomson effect. Considering that the condensation temperature of H_2, CO and CH_4 is

below −100°C, in this work, there will not be condensation of the gas at the backpressure regulator.

Before testing, 15 mL of 0.1 M $KCHO_3$ electrolyte (or other required electrolyte) was added to the cathode and anode cell body, respectively. Secondly, connecting the gas bubbling tube, working electrode, counter electrode (a graphite rod) and reference electrode (Supplementary Fig. 38) to the corresponding positions of the chamber cover. Then, the cover is tightly fixed to the cell body using 8 bolts (M10). Under stirring conditions, the gas in the CO_2 cylinder was fed into both chambers with the same flow rate (10 sccm). After keeping the gas continuously fed into the reactor for 30 minutes, the performance test is then started.

To verify that the electrolyte before the reaction could be saturated. The pH of the electrolyte in the reactor was also separately tested (Supplementary Fig. 39) using cyclic voltammetry. The result shows that the CO_2 concentration in the electrolyte had reached its saturation solubility at that temperature and pressure following our method of operation.

2. Calculations for voltage conversion

Applied cathode potentials were converted to the SHE reference scale using $E_{SHE} = E_{Ag/AgCl} + 0.197$ V.

Applied cathode potentials were converted to the RHE reference scale using $E_{RHE} = E_{Ag/AgCl} + 0.197 \text{ V} + 0.059 \times \text{pH}$. The pH used in the calculation is that of the bulk electrolyte.

3. Calculations for ^{1}H-NMR quantification.

The basis of ^{1}H-NMR is that the signal intensity in the NMR spectrum is directly proportional to the number of nuclei responsible for that particular resonance. For CH_3OH and internal standard DMSO ($(CH_3)_2SO$), it is the hydrogen nuclei on the methyl group ($-CH_3$) that generates the resonance peaks, so a methanol molecule possesses three resonantly active hydrogen nuclei (N = 3) and a DMSO molecule

possess six resonantly active hydrogen nuclei (N = 6). The molar ratio M_X/M_Y between two compounds X and Y can be calculated by employing the following expression:

$$\frac{M_X}{M_Y} = \frac{I_X}{I_Y} \times \frac{N_Y}{N_X}$$

Where I refers to the area of the resonance peak corresponding to X (Y). As an example, the ^{1}H-NMR data of CH_3OH in this work is calculated (X = CH_3OH, Y = DMSO, Supplementary Fig. 10).

In a typical procedure, 400 μL of electrolyte was mixed with 100 μL of a solution of 10 mM dimethyl sulfoxide (DMSO) and 50 mM phenol in D_2O for the ^{1}H-NMR analysis. Therefore, the amount of DMSO in the ^{1}H-NMR sample is 10^{-6} mol (10 mM × 100 μL = 10^{-6} mol). The integration operation of the resonance peaks using OriginPro 2018 Learning Edition software yields the absolute peak area corresponding to DMSO as 0.8394, and the absolute peak area corresponding to CH_3OH is 0.7075. Therefore, the amount of CH_3OH in the ^{1}H-NMR sample is:

$$M_X = M_Y\,(10^{-6}) \times \frac{I_X\,(0.70752)}{I_Y\,(0.83937)} \times \frac{N_Y\,(6)}{N_X\,(3)} = 1.69 \times 10^{-6}\ \text{mol}$$

So, the total amount of methanol is 1.69×10^{-6} / 400 uL × 15 mL = 6.34×10^{-5} mol.

4. Calculation on TOF/TON of Mo_2C/N-CNT.

Turnover frequency (TOF) is calculated using the following formula (assuming all the supported Mo_2C particles as active sites):[1]

$$\text{TOF}\,(s^{-1}) = \frac{j \times FE}{F \times \alpha \times N}$$

where N is the number of moles of Mo_2C deposited on the gas diffusion electrode (the weight percentage of Mo in the Mo_2C/N-CNT sample was ~10.77%, as confirmed by ICP-OES measurements), j is the total current density, α is the number of electrons transferred (α = 6 for reduction of CO_2 to CH_3OH), F is Faraday's constant (96485 C/mol).

Turnover number (TON) is calculated using the following formula (assuming all the

supported Mo_2C particles are counted as active sites):

$$\mathrm{TON} = \mathrm{TOF} \times t$$

where TOF is the turnover frequency, t is the measurement duration (the measurement duration to obtain the highest FE on Mo_2C/N-CNT is 10800s).

5. Aspen simulations under different pressures.

CO_2 can produce H_2CO_3 when dissolved in water and H_2CO_3 is partially ionized. ASPEN Plus can use the “ELEC Wizard” to generate a variety of electrolyte components that can be formed by various ionization reactions. The final determined components include H_2O, CO_2, OH^-, HCO_3^- and CO_3^{2-}. In the simulation, the used ASPEN Plus module is Flash2 (Supplementary Fig. 40) and the property method is chosen as ELECNRTL. The Sensitivity function was used for CO_2 solubility analysis, the pressure of the Flash2 modules is used as an argument. Compared to 1 atm (0.1 MPa), the solubility of CO_2 in water increases significantly under 40 atm (4.0 MPa) at 25 ℃.

6. Calculation on initial equilibrium concentration values of species in 0.1 M $KHCO_3$ under different pressures.

In aqueous solutions, H_2CO_3 experiences two steps of dissociations:

$$H_2CO_3 \rightleftharpoons HCO_3^- + H^+,$$

$$k_1 = ([H^+] \times [HCO_3^-]) / [H_2CO_3] = 2.5\times10^{-4}\ \mathrm{M},$$

$$HCO_3^- \rightleftharpoons CO_3^{2-} + H^+,$$

$$k_2 = ([H^+] \times [CO_3^{2-}]) / [HCO_3^-] = 4.84\times10^{-11}\ \mathrm{M}.$$

According to charge conservation in electrolyte:

$$[K^+] + [H^+] = [HCO_3^-] + 2 \times [CO_3^{2-}] + [OH^-].$$

And according to the results of Aspen simulation.

Together with the self-ionization equation of water:

$$H_2O \rightleftharpoons [H^+] + [OH^-],$$

$$k_w = [H^+] \times [OH^-] = 10^{-14},$$

The initial equilibrium concentration values of carbon species (i.e., HCO_3^-, CO_3^{2-}) and protons (i.e., pH) in 0.1 M $KHCO_3$ electrolyte under various pressures were calculated using MATLAB software. The results were summarized in Supplementary Table 11.

7. Calculation of the diffusion coefficients.

All molecular dynamics (MD) simulations were performed with Materials Studio program. Since 0.1 M $KHCO_3$ electrolyte was used during performance tests, one CO_2, two K^+, two HCO_3^- and 1111 water molecules were added into the simulation box with periodic boundary conditions. The above model construction processes were performed using an Amorphous Cell module.

In this study, COMPASS II force field was implemented during all simulation tasks.[2] The electrostatic interactions were calculated with the Ewald method and the van der Waals interactions were represented by the Lennard-Jones potential with a cutoff value of 15.5 Å.[3] The Smart algorithm was employed during the geometry optimization for models, with the cells optimized meanwhile until the convergence tolerance satisfied the criteria of energy less than 2.0×10^{-5} kcal/mol, force less than 0.001 kcal/mol/Å and displacement less than 1.0×10^{-5} Å. Then, a 500 ps equilibration at 298.15 K and 1 bar was carried out, followed by a 3 ns MD run with a time step of 1 fs under the NPT ensemble at 298.15 K and different pressure. The system temperature and pressure were controlled by the Nose thermostat and Berendsen barostat, respectively.

Finally, the MD trajectory files were used to calculate the mean square displacement (MSD), then the diffusion coefficient of species in 0.1 M $KHCO_3$ was analyzed from MSD data. MSD is calculated by the following formula.[4]

$$\mathrm{MSD}\ (t) = \frac{1}{N}\sum_{i=1}^{N}(|r_i(t+\Delta t)-r_i(t)|)^2$$

Where $r_i(t)$ is the vector position of molecule i at time t and N is the number of molecules. According to Einstein's diffusion law, the change of MSD with time

indicates the diffusion behavior of species in liquid. The diffusion coefficient is calculated by the equation.[5]

$$DC = \frac{1}{6}\frac{\partial\langle r^2(t)\rangle}{\partial t}$$

Where $\langle r^2(t)\rangle$ is the MSD and t is time.

8. Calculation for the local species concentrations.

Various equilibrium reactions in the CO_2RR system are:

$$CO_2 + OH^- \rightarrow HCO_3^- + H_2O,$$

$$K_1 = 4.44 \times 10^7 \text{ 1/M},$$

$$HCO_3^- + OH^- \rightarrow CO_3^{2-} + H_2O,$$

$$K_2 = 4.66 \times 10^3 \text{ 1/M},$$

Film theory is assumed to be applicable where, in the concentration boundary layer, the velocity gradients or convective effects are assumed to be negligible. The following balances would then occur within a slice of solution from x to x + Δx in Supplementary Fig. 41:

$$\partial[CO_2\,(aq)] / \partial t = D_{CO2} \times \partial^2[CO_2\,(aq)] / \partial x^2 - [CO_2\,(aq)] \times [OH^-] \times k_{1f} + [HCO_3^-] \times k_{1r},$$

$$\partial[HCO_3^-] / \partial t = D_{HCO3-} \times \partial^2[HCO_3^-] / \partial x^2 + [CO_2\,(aq)] \times [OH^-] \times k_{1f} - [HCO_3^-] \times k_{1r} - [HCO_3^-] \times [OH^-] \times k_{2f} + [CO_3^{2-}] \times k_{2r}$$

$$\partial[CO_3^{2-}] / \partial t = D_{CO32-} \times \partial^2[CO_3^{2-}] / \partial x^2 + [HCO_3^-] \times [OH^-] \times k_{2f} - [CO_3^{2-}] \times k_{2r},$$

$$\partial[OH^-] / \partial t = D_{OH-} \times \partial^2[OH^-] / \partial x^2 - [CO_2\,(aq)] \times [OH^-] \times k_{1f} + [HCO_3^-] \times k_{1r} - [HCO_3^-] \times [OH^-] \times k_{2f} + [CO_3^{2-}] \times k_{2r}$$

The rate constants for the forward and reverse reactions (i.e., k_{1f}, k_{1r}, k_{2f}, k_{2r}) are given in Supplementary Table 9 and diffusion coefficients (i.e., D_{CO2}, D_{HCO3-}, D_{CO32-}, D_{OH-}) at different pressures for various species at 25 °C are listed in Supplementary Table 10.

According to the Supplementary Table 11 and performance test, the rate for CO_2 consumption (i.e., CO_2RR) and OH^- formation on the electrode surface can be

calculated as:

$$CO_{2consumption} = (j / F) \times (FE_{HCOO^-} / N_{HCOO^-} + FE_{CO} / N_{CO} + FE_{CH4} / N_{CH4} + FE_{CH3OH} / N_{CH3OH})$$

$$OH_{formation} = (j / F) \times (FE_{HCOO-} / N_{HCOO-} + 2 \times FE_{CO} / N_{CO} + 8 \times FE_{CH4} / N_{CH4} + 6 \times FE_{CH3OH} / N_{CH3OH} + 2 \times FE_{H2} / N_{H2})$$

j is the geometric current density of Mo_2C/N-CNT in A/m^2.

The above equations are second-order time-dependent partial differential equations that are to be solved under the following boundary conditions. The initial values of the concentrations (at t=0, before current flows) are assumed to be the same in the bulk solution and are listed in Supplementary Table 12 for different pressures.

At time t > 0 and x = 0 (i.e., at the interface of bulk solution and the boundary layer):

$$[CO_2\,(aq)] = [CO_2\,(aq)]_B$$

$$[HCO_3^-] = [HCO_3^-]_B$$

$$[CO_3^{2-}] = [CO_3^{2-}]_B$$

$$[OH^-] = [OH^-]_B$$

where $[CO_2\,(aq)]_B$, $[HCO_3^-]_B$, $[CO_3^{2-}]_B$, and $[OH^-]_B$ are the equilibrium values in the bulk solution, and so the same as the values given in Supplementary Table 11.

At time t > 0 and x = δ (i.e., at the electrode surface) are related to the reaction fluxes (i.e., the electrode surface is an impermeable, reflective wall for all species):

$$D_{CO2} \times d[CO_2\,(aq)] / dx = -CO_{2consumption}$$

$$D_{HCO3-} \times d[HCO_3^-] / dx = 0$$

$$D_{CO32-} \times d[CO_3^{2-}] / dx = 0$$

$$D_{OH-} \times d[OH^-] / dx = OH_{formation}$$

With all the boundary conditions and constants known, the partial differential equations were solved using MATLAB software.

References

1. Wu Y, Jiang Z, Lu X, Liang Y, Wang H. Domino Electroreduction of CO_2 to Methanol on a Molecular Catalyst. Nature **2019**, 575, 639-642.
2. Sun H, et al. COMPASS II: Extended Coverage for Polymer and Drug-like Molecule Databases, J. Mol. Model. **2016**, 22, 1-10.
3. Lennard-Jones JE. Cohesion. Proc. Phys. Soc. **1931**, 43, 461-482.
4. Akbarzadeh H, Yaghoubi H. Molecular Dynamics Simulations of Silver Nanocluster Supported on Carbon Nanotube. J. Colloid Interface Sci. **2014**, 418, 178-184.
5. Frenkel D, Smit B. Chapter 5 - Monte Carlo Simulations in Various Ensembles. Understanding Molecular Simulation (Second Edition). Academic Press: San Diego, **2002**, 111-137.
6. Li J, et al. Electroreduction of CO_2 to Formate on a Copper-Based Electrocatalyst at High Pressures with High Energy Conversion Efficiency. J Am Chem Soc. **2020**, 142(16): 7276-7282.
7. Boutin E, et al. Aqueous Electrochemical Reduction of Carbon Dioxide and Carbon Monoxide into Methanol with Cobalt Phthalocyanine. Angew. Chem. Int. Ed. **2019,** 58 (45), 16172-16176.
8. Zhang W, et al. Electrochemical Reduction of Carbon Dioxide to Methanol on Hierarchical Pd/SnO_2 Nanosheets with Abundant Pd-O-Sn Interfaces. Angew. Chem. Int. Ed. **2018,** 57 (30), 9475-9479.
9. Mou S, et al. Boron Phosphide Nanoparticles: A Nonmetal Catalyst for High-Selectivity Electrochemical Reduction of CO_2 to CH_3OH. Adv. Mater. **2019,** 31 (36), e1903499.
10. Periasamy AP, et al. Facet- and Structure-dependent Catalytic Activity of Cuprous Oxide/Polypyrrole Particles towards the Efficient Reduction of Carbon Dioxide to Methanol. Nanoscale **2018,** 10 (25), 11869-11880.
11. Low QH, Loo NWX, Calle-Vallejo F, Yeo BS. Enhanced Electroreduction of Carbon Dioxide to Methanol Using Zinc Dendrites Pulse-Deposited on Silver Foam. Angew. Chem. Int. Ed. **2019,** 58 (8), 2256-2260.
12. Irfan Malik M, Malaibari ZO, Atieh M, Abussaud B. Electrochemical Reduction of CO_2 to Methanol over MWCNTs Impregnated with Cu_2O. Chem. Eng. Sci. **2016,** 152, 468-477.
13. Huang J, Guo X, Yue G, Hu Q, Wang L. Boosting CH_3OH Production in Electrocatalytic CO_2 Reduction over Partially Oxidized 5 nm Cobalt Nanoparticles Dispersed on Single-Layer Nitrogen-Doped Graphene. ACS Appl. Mater. Interfaces **2018,** 10 (51), 44403-44414.
14. Ji L,et al. Highly Selective Electrochemical Reduction of CO_2 to Alcohols on an FeP Nanoarray. Angew. Chem. Int. Ed. **2020,** 59 (2), 768-772.
15. Lv J-J, et al. A Highly Porous Copper Electrocatalyst for Carbon Dioxide Reduction. Adv. Mater. **2018,** 30 (49), e1803111.
16. Burdyny T, et al. Nanomorphology-Enhanced Gas-Evolution Intensifies CO_2 Reduction Electrochemistry. ACS Sustain. Chem. Eng. **2017,** 5 (5), 4031-4040.
